# The elasticity of silicone-stabilized liposomes has no impact on their in vivo behavior

**DOI:** 10.1186/s12951-024-02698-9

**Published:** 2024-08-05

**Authors:** Alicja Hinz, Joanna Lewandowska-Łańcucka, Ewa Werner, Agnieszka Cierniak, Krystyna Stalińska, Grzegorz Dyduch, Michał Szuwarzyński, Monika Bzowska

**Affiliations:** 1 Department of Cell Biochemistry, Faculty of Biochemistry, Biophysics and Biotechnology, Jagiellonian University, Gronostajowa 7, Kraków, 30-387 Poland; 2 Department of Physical Chemistry, Faculty of Chemistry, Jagiellonian University, Gronostajowa 2, Kraków, 30- 387 Poland; 3 Animal Facility, Faculty of Biochemistry, Biophysics and Biotechnology, Jagiellonian University, Gronostajowa 7, Kraków, 30-387 Poland; 4 Department of Biochemistry, Faculty of Medicine and Health Sciences, Andrzej Frycz Modrzewski Krakow University, Gustawa Herlinga-Grudzińskiego 1, Kraków, 30-705 Poland; 5 Department of Pathomorphology, Jagiellonian University Medical College, Grzegórzecka 16, Kraków, 33-332 Poland; 6 Academic Centre for Materials and Nanotechnology, AGH University of Krakow, Al. Mickiewicza 30, Krakow, 30-059 Poland

**Keywords:** Liposomes, Silicone network, Nanocarrier elasticity, Nanoparticles biodistribution, Nanotoxicity, Proinflammatory cytokines, Nanoparticles tumor accumulation

## Abstract

**Background:**

The elastomechanical properties of nanocarriers have recently been discussed as important for the efficient delivery of various therapeutics. Some data indicate that optimal nanocarriers’ elasticity can modulate in vivo nanocarrier stability, interaction with phagocytes, and uptake by target cells. Here, we presented a study to extensively analyze the in vivo behavior of LIP-SS liposomes that were modified by forming the silicone network within the lipid bilayers to improve their elastomechanical properties. We verified liposome pharmacokinetic profiles and biodistribution, including retention in tumors on a mouse model of breast cancer, while biocompatibility was analyzed on healthy mice.

**Results:**

We showed that fluorescently labeled LIP-SS and control LIP-CAT liposomes had similar pharmacokinetic profiles, biodistribution, and retention in tumors, indicating that modified elasticity did not improve nanocarrier in vivo performance. Interestingly, biocompatibility studies revealed no changes in blood morphology, liver, spleen, and kidney function but indicated prolonged activation of immune response manifesting in increased concentration of proinflammatory cytokines in sera of animals exposed to all tested liposomes.

**Conclusion:**

Incorporating the silicone layer into the liposome structure did not change nanocarriers’ characteristics in vivo. Further modification of the LIP-SS surface, including decoration with hydrophilic stealth polymers, should be performed to improve their pharmacokinetics and retention in tumors significantly. Activation of the immune response by LIP-SS and LIP-CAT, resulting in elevated inflammatory cytokine production, requires detailed studies to elucidate its mechanism.

**Graphical Abstract:**

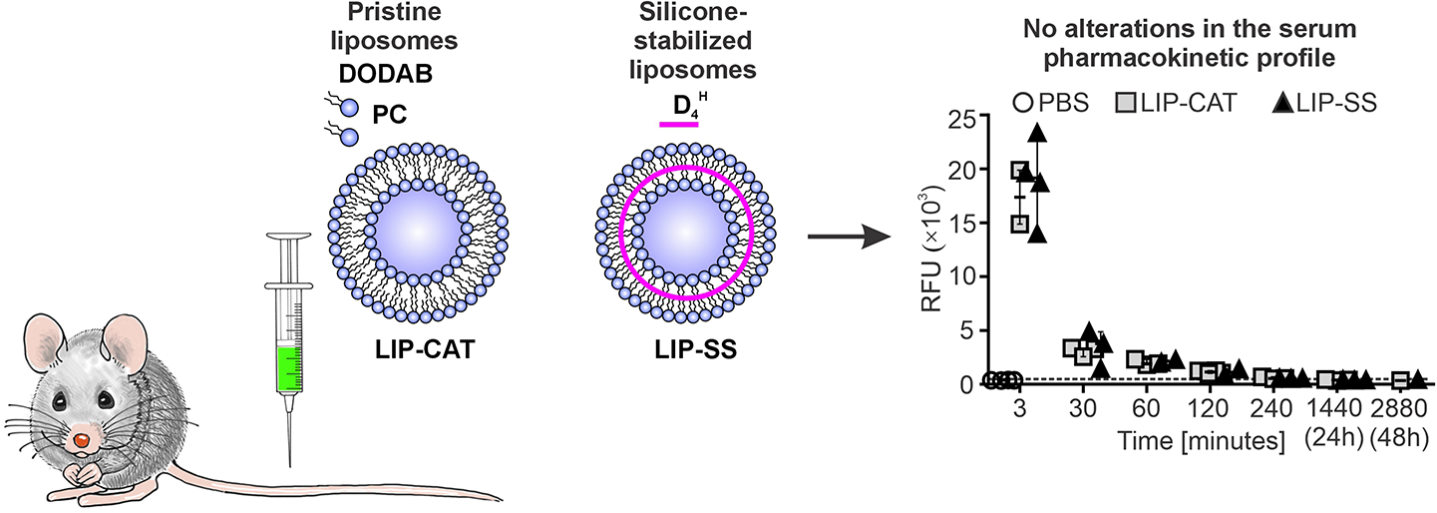

**Supplementary Information:**

The online version contains supplementary material available at 10.1186/s12951-024-02698-9.

## Background

Lipid nanoparticles, primarily liposomes, are the most critical nanomedicines due to their biocompatibility, uncomplicated and low-cost synthesis, and ability to encapsulate chemically diverse molecules. Since the U.S. Food and Drug Administration (FDA) approved Doxil, the first liposomal drug, in 1995, the pool of liposome- or lipid-based nanoparticles has grown to more than 30, and the molecules delivered include anticancer drugs, immunosuppressive drugs, and mRNA encoding viral proteins [1, 2]. Despite many years of clinical use, liposomes are still not considered optimal nanocarriers, and their main drawbacks are insufficient physical, chemical, and biological stability, including a tendency to aggregate and uncontrolled drug release [3]. Modifying the liposome chemical composition is the most common action aimed primarily at improving liposome behavior in vivo. Covalent cross-linking of the lipid bilayer, cholesterol, or chitosan incorporation into a lipid bilayer, but predominantly, surface modification with polyethylene glycol (PEG), can markedly improve liposome stability in blood circulation, and thus delivery to sick tissue [4–7]. However, recent data from animal studies and clinical observations revealed that PEGylated nanomedicines might induce an immune response that involves the production of anti-PEG antibodies, activation of the complement system, and hypersensitivity reactions [8]. It is, therefore, justified to develop alternative methods to improve the in vivo performance of liposomes.

Recently published observations indicate optimal elastomechanical properties of nanoparticles might be important for their stability in vivo and uptake by phagocytic or target cells (i.e., tumor cells). Yi et al. and Shen et al. presented the results of computer simulations showing that soft nanoparticles, when interacting with the cell membrane, can stick and flatten on its surface. Consequently, the convex cell membrane surrounds them faster in the first uptake phase. Subsequently, the absorption process of soft nanoparticles is slowed down, and eventually, a faster uptake of rigid nanomaterials is observed [9, 10]. The phenomenon of far more rapid cellular uptake of rigid than softer nanoparticles’ was presented by Hui et al. Using the library of silica nanocapsules differing in Young’s modules, the authors observed that the differences in cellular uptake depending on elastic modules are more evident for phagocytic cells (e.g., RAW264.7, mouse monocyte/macrophage cell line) than for cancer cells (SKOV3, human ovarian cancer cell line) and suggested that this phenomenon might be related to the different mechanisms of endocytosis of materials found in these cells [11]. Ma et al. also indicated that RAW 264.7 and HeLa (human adenocarcinoma cell line derived from cervical epithelial cells) were more likely to take up the stiff SiO_2_ capsule than the softer ones. They confirmed that the uptake mechanism for stiff SiO_2_ capsules is clathrin-mediated, but for softer SiO_2_ capsules, it is *via* a caveola-dependent pathway [12]. Interestingly, another team presented results indicating that RAW 264.7 cells take up rigid hydrogel-based nanoparticles *via* the clathrin-dependent pathway, while nanoparticles with lower stiffness due to macropinocytosis. However, they did not confirm that the uptake intensity depended on the elasticity features [13]. On the other hand, Teng et al. showed that MCF-7 cells (human breast adenocarcinoma cell line) took up softer silica nanoparticles more easily than stiff ones [14]. Nanoparticle elasticity also seems essential for improving nanocarriers’ in vivo fate. Anselmo et al. showed that a more significant amount of softer PEG-based hydrogel nanoparticles than stiff nanoparticles circulated in the blood after intravenous injection. However, this phenomenon was observed only up to 2 h after administration of nanoparticles. Subsequently, the differences in pharmacokinetic profiles between soft and stiff nanoparticles were considered negligible [15]. Yet other studies performed for small unilamellar vesicles differing in elastomechanical properties demonstrated that in vivo tumor penetration might vary depending on vesicle rigidity [16]. In light of these observations, it seems necessary to conduct further studies that will more closely verify the dependence between the elasticity of nanocarriers and the efficiency of their tumor accumulation and uptake by different cells.

In our previous studies, we obtained and characterized novel liposomes stabilized with a silicone layer. Developed by us, the stabilization method allowed us to obtain stable nanocarriers with the zeta potential values of the pristine liposomes. The protocol involved the base-catalyzed polycondensation of commercially available silicone precursor – 1,3,5,7-tetramethylcyclotetrasiloxane (D_4_^H^) occurring within the liposomal bilayer. Transmission electron microscopy (TEM) and dynamic light scattering (DLS) measurements demonstrated that the modified silicone liposomes exhibit the typical lipid vesicle’s size and morphology. However, their stability in vitro is significantly improved. We demonstrated that silicone-stabilized liposomes have lower calcein permeability than pristine liposomes after Triton X-100 titration. Moreover, fetal bovine serum had no noticeable effect on the permeability of the tested liposome membrane to calcein [17].

We also investigated elastomechanical features since they are essential when considering carrier-cell interactions. Using atomic force microscopy (AFM) measurements and applying the Derjaguin–Muller–Toporov (DMT) model, the elastic modulus of the silicone-stabilized and the pristine liposomes was assessed. Our findings revealed that the silicone network formed inside the liposomes bilayer resulted in higher values of DMT modulus (35 GPa and 5 MPa for stabilized and pure vesicles, respectively) and, therefore, increased the rigidity of these nanocarriers compared to pristine ones. Importantly, high biocompatibility of stabilized liposomes in vitro was also demonstrated [18]. In this work, we focused for the first time on the in vivo behavior of intravenously administered silicone-stabilized liposomes. We used liposomes with identical surfaces despite using a silicone layer for stabilization. Hence, they were a perfect model to study the effect of liposomes’ exclusively elastomechanical properties on their behavior in vivo. First, we were interested in verifying whether improving the elastomechanical properties of liposomes would be sufficient to obtain long-circulating liposomes without needing, i.e., PEG functionalization. The pharmacokinetics, biodistribution, and elimination routes of the tested nanocarriers were performed in a mouse model of breast cancer to investigate simultaneously their tumor localization. Additionally, we evaluated the acute and long-term toxicity of silicone-stabilized liposomes in healthy mice. Our results are significant in light of understanding the influence of the elastomechanical properties of liposomes on their biological behavior.

## Materials and methods

### Materials

1,3,5,7-Tetramethylcyclotetrasiloxane (D_4_^H^, ABCR), L-α-phosphatidylcholine from frozen egg yolk type XVI-E (PC lipid, Sigma, 100 mg/ml solution in ethanol), dimethyldioctadecylammonium bromide (DODAB, Fluka), L-α-Phosphatidylethanolamine-N-(lissamine rhodamine B sulfonyl) (Ammonium Salt) (PE-Rhod) (Avanti). Lipopolysaccharide (LPS) from Escherichia coli 0111:B4, flagellin from Bacillus subtilis, FSL-1, and CU-T12-9 were from InvivoGen, San Diego, CA, USA. Formaldehyde solution 4%, buffered, pH 6.9, and eosin Y alcoholic solution, with phloxine, were from Merck (Darmstadt, Germany). The hematoxylin stain solution modified Harris’ formulation, and xylene was from VWR International (Radnor, U.S.).

### Preparation of cationic (LIP-CAT) and silicone-stabilized (LIP-SS) liposomes

The liposomes were obtained utilizing the procedure we developed and described earlier [18]. For LIP-CAT, 50 µl of DODAB solution (8.4 mg/ml in ethanol) was mixed with 50 µl of PC solution (100 mg/ml in ethanol). Such an amount of DODAB constituted 10% of PC molar content. The mixture was vortexed for about 5 min, and ethanol was evaporated under the gentle stream of nitrogen. Thus, the obtained film was subsequently hydrated with 5 ml of PBS and vortexed. The resulting multilamellar liposomal dispersion was extruded five times through the membrane filters with 100-nm pores using a gas-pressurized extruder. The silicone-stabilized liposomes (LIP-SS) were fabricated by adding to the ethanolic solutions of PC and DODAB an appropriate amount of D_4_H (1 mg), which constituted 60% of the total molar content of used lipids. Then, the protocol for LIP-CAT preparation was applied. To initiate the polycondensation processes of the precursor, the resulting film was hydrated with PBS at pH adjusted to 8.5 (this PBS was used as a control in all biological experiments). After extrusion, the dispersion was stirred for 24 h at room temperature.

### Preparation of fluorescently labeled liposomes LIP-CAT_Rho_ and LIP-SS_Rho_

Fluorescently labeled liposomes were obtained using the procedure described above for the preparation of liposomes, with the only difference being that to the ethanolic solution of PC, DODAB (LIP-CAT), and D_4_^H^ (LIP-SS), the appropriate amount of the PE-Rhod stock solution (1 mg/ml in ethanol) was added. The PE-Rhod concentration in the liposome dispersions was optimized to achieve a high fluorescence signal while minimizing the impact on the size of the resulting labeled nanocarriers. The selected concentration was PE-Rhod = 5 × 10^− 6^ M. The fluorescence spectra of the samples (LIP-CAT_Rho_ and LIP-SS_Rho_) were recorded using the F-2700 Hitachi fluorescence spectrophotometer at the excitation wavelength of 560 nm (see Figure S1C).

### Characterization of resulting liposomes

The obtained liposomes (LIP-CAT, LIP-SS, LIP-CAT_Rho_, LIP-SS_Rho_) were characterized for their hydrodynamic diameter and zeta potential values using a Malvern Nano ZS light-scattering apparatus (Malvern Instrument Ltd., Worcestershire, UK). The time-dependent autocorrelation function of the photocurrent was acquired every 10 s, with 15 acquisitions for each run. The sample of solutions was illuminated by a 633 nm laser, and the intensity of light scattered at an angle of 173º was measured by an avalanche photodiode. The z-averaged hydrodynamic mean diameters (dz) and dispersity index (DI) of the samples were calculated using the software provided by Malvern. The zeta potential of liposomes was measured using the technique of Laser Doppler Velocimetry (LDV). Each value was obtained as an average from three runs with at least 10 measurements. Data are presented as Mean ± SD (see Figure S1B in Supplementary Materials). The liposomes concentration (LIP-CAT and LIP-SS) determined using an LM10 Nanosight instrument (Malvern Instruments Ltd) equipped with an sCMOS camera (Hamamatsu Photonics, Hamamatsu, Japan) and a 450 nm blue laser was in the range of 10^12^ particles/ml. Cryogenic Transmission Electron Microscopy (cryo-TEM) images of developed samples were collected with a Glacios Cryo-TEM microscope (Thermo Fisher Scientific) at an accelerating voltage of 200 kV with a Falcon 4 Thermo Fisher Scientific detector. The samples (about 3 µl) were deposited on freshly glow-discharged TEM grids (Quantifoil R2/1, Cu, mesh 200) and plunged-frozen in liquid ethane utilizing a Vitrobot Mark IV (Thermo Fisher Scientific). Frozen grids were next clipped in liquid nitrogen and loaded into the microscope. The images were recorded at microscope magnification ranging from 100 000 to 120 000x. The resulting images are depicted in Figure S1A in Supplementary Materials. The diameter of liposomes calculated based on cryo-TEM images are presented in Figure S1B (Supplementary Materials).

Atomic Force Microscopy topography and Derjaguin–Muller–Toporov (DMT) modulus images (see Figure S2) were obtained with a Dimension Icon XR atomic force microscope (Bruker, Santa Barbara, CA, USA) working in the PeakForce Quantitative Nanomechanical Mapping (PF QNM) mode in the air. DMT model was used to determine nanomechanical parameters of the obtained LIP-CAT, LIP-SS, LIP-CAT_Rho_, and LIP-SS_Rho_ liposomes. LIP-CAT or LIP-CAT_Rho_ samples were measured with calibrated silicon probes (Bruker) with a spring constant of 0.35 N/m, tip radius of 5 nm, triangular geometry, and working with the resonance frequency of 67 kHz. LIP-SS or LIP-SS_Rho_ samples were measured with another type of calibrated probe (Bruker) with a spring constant of 202 N/m, tip radius of 12 nm, rectangular geometry, and working resonance frequency of 472 kHz. The probe calibration process was done after the PF-QNM Manual (Bruker). Deflection sensitivity data were obtained by engaging and ramping the probe onto a hard sapphire surface, and spring constant values were calculated after a thermal tuning process. For the measurements, all liposomes were deposited on the flat silicon surfaces (ON Semiconductor, Czechia) of crystallographic orientation of 〈100〉 previously purified in a “piranha” solution (a mixture of H_2_SO_4_ and H_2_O_2_ at a 3:1 ratio) using a drop-casting technique. After one hour of deposition, surfaces were dried in the pure air stream.

### Cells used in experiments

Murine mammary carcinoma 4T1 cells stably expressing firefly luciferase were purchased from Dr. Gary Sahaian’s lab (Tufts University, MA, Boston). Human TLR4/NF-ĸB-SEAP reporter HEK293 cells, human TLR5/NF-ĸB-SEAP reporter HEK293 cells, human TLR2 + TLR1/NF-ĸB-SEAP reporter HEK293 cells and human TLR2 + TLR6/NF-ĸB-SEAP reporter HEK293 cells were purchased from InvivoGen (San Diego, CA, USA). Murine monocyte/macrophage RAW 264.7 cells (ATCC TIB-71) were obtained from the American Type Culture Collection (Manassas, VA, USA). All cells were grown in Dulbecco’s modified Eagle’s medium (DMEM) containing 4.5 g/l glucose (GIBCO, Paisley, UK) and 10% (v/v) fetal bovine serum (FBS, GIBCO, Paisley, UK).

### Detection of LPS contamination in liposome

Detection of LPS in liposome samples was analyzed using Pierce Chromogenic Endotoxin Quant Kit (ThermoFisher Scientific, Rochester, NY, USA) according to the manufacturer’s procedure. The standard curve was performed based on Low Standards (0.1-1 EU/ml of lyophilized endotoxin). Tested liposome solutions were diluted 100 times. The absorbance of tested samples was measured at 405 nm using a microplate reader Synergy H1 Hybrid plate reader controlled by Gene5 version 2.00.18 software (BIOTEK Instruments, Winooski, VT, USA).

### Analysis of nitrate oxide (NO) production by RAW 264.7 cells

The liposome effect on NO production by murine monocyte-macrophage cells RAW 264.7 was analyzed using the Griess assay. The cells were grown overnight in DMEM enriched with 10% FBS on a 96-well plate (at the density of 1 × 10^4^ cells per well). Next, the medium was replaced with 100 µl of fresh medium (DMEM, 2% FBS) supplemented with: (1) LPS (100 ng/ml), (2) IFN-γ (10 ng/ml), (3) LPS (100 ng/ml) + IFN-γ (10 ng/ml), 3) LIP-CAT 10 µl, (4) LIP-SS 10 µl, (5) LIP-CAT 10 µl + LPS (100 ng/ml), (6) LIP-SS 10 µl + LPS (100 ng/ml), (7) LIP-CAT 10 µl + IFN-γ (10 ng/ml), 4) LIP-SS 10 µl + IFN-γ (10 ng/ml). Control cells (negative control) were treated with a medium containing 10 µl of PBS. The cells were grown for 24 h, and then the cultured media were collected and transferred to the fresh 96-well plate. NO level in the collected media was analyzed using the Griess reaction using the procedure described by us previously [19].

### Liposome interaction with TLR receptors

The interaction of liposomes with selected TLR receptors was analyzed by measurement of SEAP (secreted embryonic alkaline phosphatase) activity secreted to the culture media of reporter HEK293 cells. Human TLR4/NF-ĸB-SEAP, TLR5/NF-ĸB-SEAP, TLR2 + TLR1/NF-ĸB-SEAP and TLR2 + TLR6/NF-ĸB-SEAP reporter HEK293 cells were seeded on 96-well plate at the density of 25 × 10^3^ in 100 µl of DMEM containing 10% v/v of FBS and antibiotics (100 U/ml penicillin and 100 µg/ml streptomycin). Next, 10 µl of PBS, LIP-CAT, or LIP-SS were added to the medium. Additionally, positive controls were prepared by adding ligands specific for specific TLRs (10 ng/ml LPS for TLR4, 2 µM CU-T12-9 for TLR2/TLR1 heterodimer, 1 ng/ml FSL-1 for TLR2/TLR6 heterodimer and 1 ng/ml flagellin for TLR5). In this experiment, we additionally used PBS_Lonza_, i.e., PBS used for synthesizing liposomes before pH adjustment (LPS contamination measured for this solution was below the detection limit of the LAL test, < 0.01 EU/ml). After 24 h incubation, 10 µl of culture medium was collected from each well, transferred into a 96-well plate, and mixed with 90 µl of QUANTI Blue™ (InvivoGen, San Diego, CA, USA) to measure the activity of SEAP. The reaction was performed for 1 h at 37^°^C, and then the absorbance was measured at 620 nm using a Synergy H1 Hybrid microplate reader.

### Animals used in the studies

Six-week-old BALB/c female and male mice were purchased from Janvier Labs (France) and delivered by Vivari Ewa Głowacka, Regina Nowak (Poland). Mice were housed under controlled conditions and provided food and water *ad libitum*.

### Pharmacokinetic, biodistribution studies and calculations of elimination half-life of liposomes

The pharmacokinetics and biodistribution of fluorescently labeled liposomes were analyzed on mice with 4T1 breast carcinoma. Based on our previous studies, we chose an orthotopic breast tumor model based on 4T1 cells. This model allows the formation of blood vessels and a high content of collagen fibers, miming the environment of breast tumors that spontaneously develop [20]. 4T1 cells were trypsinized, collected from the dishes, centrifuged, and resuspended in sterile PBS (Lonza, Basel, Switzerland). 5 × 10^5^ cells (in 0.1 ml) were injected orthotopically into the fourth mammary fat pad of BALB/c mice. Six days later, mice were weighed and randomly divided into three experimental groups (PBS, LIP-CAT_Rho,_ or LIP-SS_Rho_) and injected intravenously (100 µl per 10 g of body weight) with PBS (control of autofluorescence), LIP-CAT_Rho_, or LIP-SS_Rho_. Before experiments, the fluorescence intensity of two different types of liposomes was confirmed to be comparable. The animals were then placed in metabolic cages for single mice (Tecniplast, Italy) to enable the collection of urine and feces. Next, at different time points after administration, animals were euthanized. Blood was collected by cardiac puncture and left for clotting; then, serum was isolated by centrifugation. Bile was isolated by puncture of the gall bladder. Organs (liver, kidneys, and spleen) and tumors were isolated, weighed, and homogenized in PBS (1 ml per 0.1 g of tissue) using gentleMACS™ M tubs and gentleMACS™ Dissociator (Miltenyi Biotec, Germany). Fluorescence measurements (560 nm excitation, emission 590 nm) were performed using Synergy H1 hybrid reader and Gene Software (Biotek Instruments, Winooski, VT, USA). The following samples (100 µl) were analyzed: serum, homogenized tissues, tumors, urine, bile (diluted 1:100 in PBS), and feces (resuspended in PBS, 1 ml per 0.1 g of feces). For biodistribution studies, % of initial dose was defined. This parameter corresponded to the total fluorescence calculated as the sum of all the fluorescence values measured for total serum, bile, organs, and tumor immediately 3 min after liposome injection. Background fluorescence measured for samples collected from control mice injected with PBS was subtracted from each sample. Raw data generated for these experiments are available in the RODBUK repository (information in section Availability of data and materials). Liposome elimination half-life calculations were based on the assumption of a first-order reaction and in accordance with Applied Biopharmaceutics & Pharmacokinetics, Chap. 2. Mathematical Fundamentals in Pharmacokinetics.

### Toxicity studies - liposomes administration and animal material isolation

Six-week-old BALB/c mice (female or male) were weighted and randomly divided into 3 experimental groups. Animals were injected intravenously with PBS, LIP-CAT, or LIP-SS (day 0 of the experiment, 100 µl of liposomes or PBS per 10 g of body weight). During the toxicity studies, animals received eight doses of PBS or liposomes administered twice weekly. The health of mice during this procedure was closely monitored. Animals were euthanized one day after the last dose of liposomes/PBS (on the 30th day of the experiment) or 30 days after the last dose of liposomes/PBS (on the 60th day of the experiment). Blood for blood morphology analysis was taken from the facial vein, and blood for cytokine profiling and biochemical parameters was obtained by cardiac puncture. Bone marrow cells were isolated by flushing the femurs with PBS.

### Hematological and biochemical analysis

The hematology analyzer ABC (Horiba, UK) allowed the analysis of the blood morphology. Biochemical markers of hepatotoxicity or nephrotoxicity were determined using Spotchem EZ Chemistry Analyzer (Woodley) and strips Spotchem Multi PANEL-V2, according to the procedure proposed by the manufacturer.

### Cytokine profiling

Isolated sera were used to analyze the concentration of selected cytokines (IL-1α, IL-1β, IL-6, IL-10, IL-12p70, IL-17 A, IL-23, IL-27, MCP-1, IFN-β, IFN-γ, TNFα, and GM-CSF). We used the LEGENDplex Mouse Inflammation Panel kit (Biolegend, San Diego, CA, USA) and the BD LSRFortessa Cell Analyzer flow cytometer (BD Bioscience, Franklins Lake, NJ, USA). The results were analyzed using LEGENDplex software (Biologend, San Diego, CA, USA).

### Analysis of the morphology of isolated tissues and SEM observation

Selected organs (liver, kidneys, and spleen) were fixed in 4% paraformaldehyde, embedded in paraffin, sectioned into slices (3 μm-thin slices), stained with hematoxylin/eosin and observed under a Leica DM6B microscope. The unstained fixed organs’ microstructure was evaluated using the cold field emission scanning electron microscope (SEM) HITACHI S-4700 with a NORAN Vantage energy dispersion spectrometer (EDS). For SEM observation, tissues were sectioned into 10 μm-thin slices and placed on the microscope slides. To remove paraffin, the samples were immersed in hexamethyldisilizane (HMDS, Sigma-Aldrich) for 10 min and finally air-dried at room temperature. Obtained materials were next stuck on the carbon tape and sputtered with a thin film of gold.

### Comet assay to analyze DNA damage in cells isolated from bone marrow

DNA damage analysis based on comet assay was performed according to the procedure described by us previously [19]. Each sample containing 1 × 10^5^ was resuspended in 100 µl of PBS. The DNA percentage in the comet tail (% DNA damage) was analyzed using an epifluorescence microscope (Olympus IX-50, Olympus Corporation, Tokyo, Japan) for two slides per sample. The analysis included 50–70 randomly selected cells from each slide.

### Statistical analysis

The obtained data were subjected to statistical analysis by determining the average of the obtained experimental points (n) along with the standard deviation (SD) using the GraphPad Prism 10 program. The number of “n” in biodistribution and pharmacokinetic analysis were different (1–4 repeats) due to the technical limitation (not enough tissue to homogenize; the mice urinated and defecated spontaneously, and the time of urination and defecation differed between animals; empty gallbladders prevented bile collection; insufficient amount of serum for analysis). We assumed that our data concerning biodistribution and pharmacokinetic analysis did not qualify for the correct application of the statistical significance test. Therefore, we assess biological significance based on the mean ± SD. Raw datasets available in the RODBUK repository: 10.57903/UJ/WTMVKS provide information about the “n” for subsequent experiments.

In toxicity studies, statistical significance was determined using the two-way repeated measures (RM) ANOVA with Geisser-Greenhouse correction followed by Tukey’s multiple comparisons test. In case of missing values within groups (resulting from cytokine level below the detection limit), a mixed-effects model (REML) was used instead of RM ANOVA. If there were missing values for at least one group (when cytokine levels for all sera were below the detection limit), statistical significance analysis could not be performed.

## Results

### LIP-SS_Rho_ liposomes have identical pharmacokinetics, biodistribution, and elimination routes as LIP-CAT_Rho_

We characterized obtained liposomes and proved the differences in their Derjaguin–Muller–Toporov modulus (29 GPa and 5 MPa for stabilized and pure vesicles, respectively). The results of liposome size and calculated modulus parameters are presented in Figures S1 and S2. The cryo-TEM images confirmed the formation of spherical structures with distinct bilayered phospholipid membrane surrounding an aqueous core in all studied samples. It seems that in all cases, the morphology of the objects observed is quite similar. The unilamellar vesicles constitute the main population. However, some multilayered liposomes have also been observed (see Figure S1A). In the case of silicone-stabilized systems, the small population of solid particles originating from the formation of monomer droplets (o/w emulsion) can be noticed. Importantly, the micrographs confirm that stabilization with the silicone layer at applied conditions did not cause the disintegration of liposomes since there were no bilayer fragments/stacks revealed for LIP-SS and LIP-SS_Rho_ samples. The mean diameters of the objects calculated based on cryo-TEM images are in the range of 76–89 nm and these values are lower than that determined by DLS measurements (see Figure S1B in Supplementary materials). Such discrepancies among the particle sizes obtained by DLS and cryo-TEM resulted from the differences in these experimental techniques. Usually, the mean size (diameter) of the particles observed on TEM micrographs is lower than that estimated from DLS measurements. The reason is that the DLS method measures the mean hydrodynamic diameter, which is heavily weighted toward the most significant structures in the solution. Due to the intensity of the scattered light, which increases with the increasing size of objects, the z-averaged hydrodynamic mean diameter obtained by cumulant analysis is overestimated [17].

**Fig. 1:**
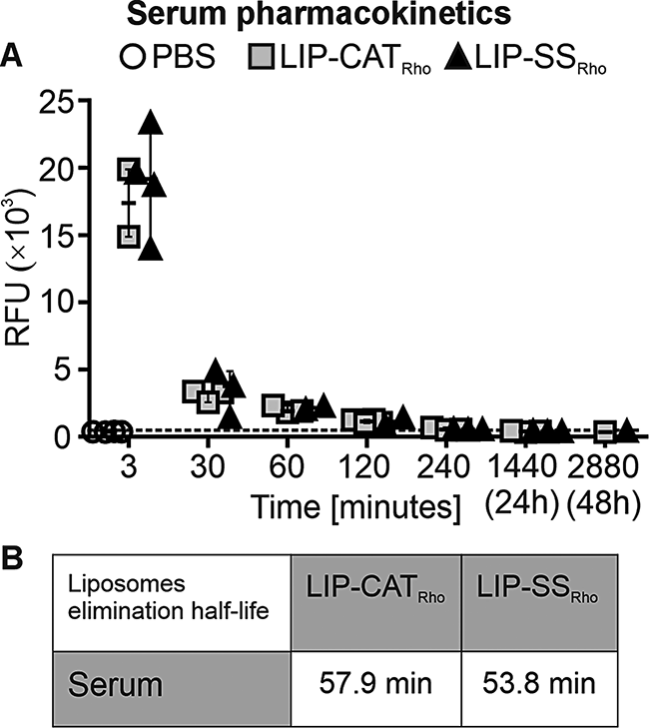
LIP-SS_Rho_ have identical serum pharmacokinetics as LIP-CAT_Rho_. Fluorescently labeled LIP-CAT_**Rho**_, LIP-SS_**Rho**_, or PBS (negative control) were injected intravenously into the BALB/c mice with orthotopic breast cancer. Animals were euthanized at different time points after injection, and blood was taken for analysis by cardiac puncture. Fluorescence measurements were performed for separated sera using a microplate reader (excitation 560 nm; emission 590 nm). (**A**) Pharmacokinetic profiles. (**B**) The serum elimination half-lives. Points represent the data obtained in different experiments (*n* = 1–4)

**Fig. 2:**
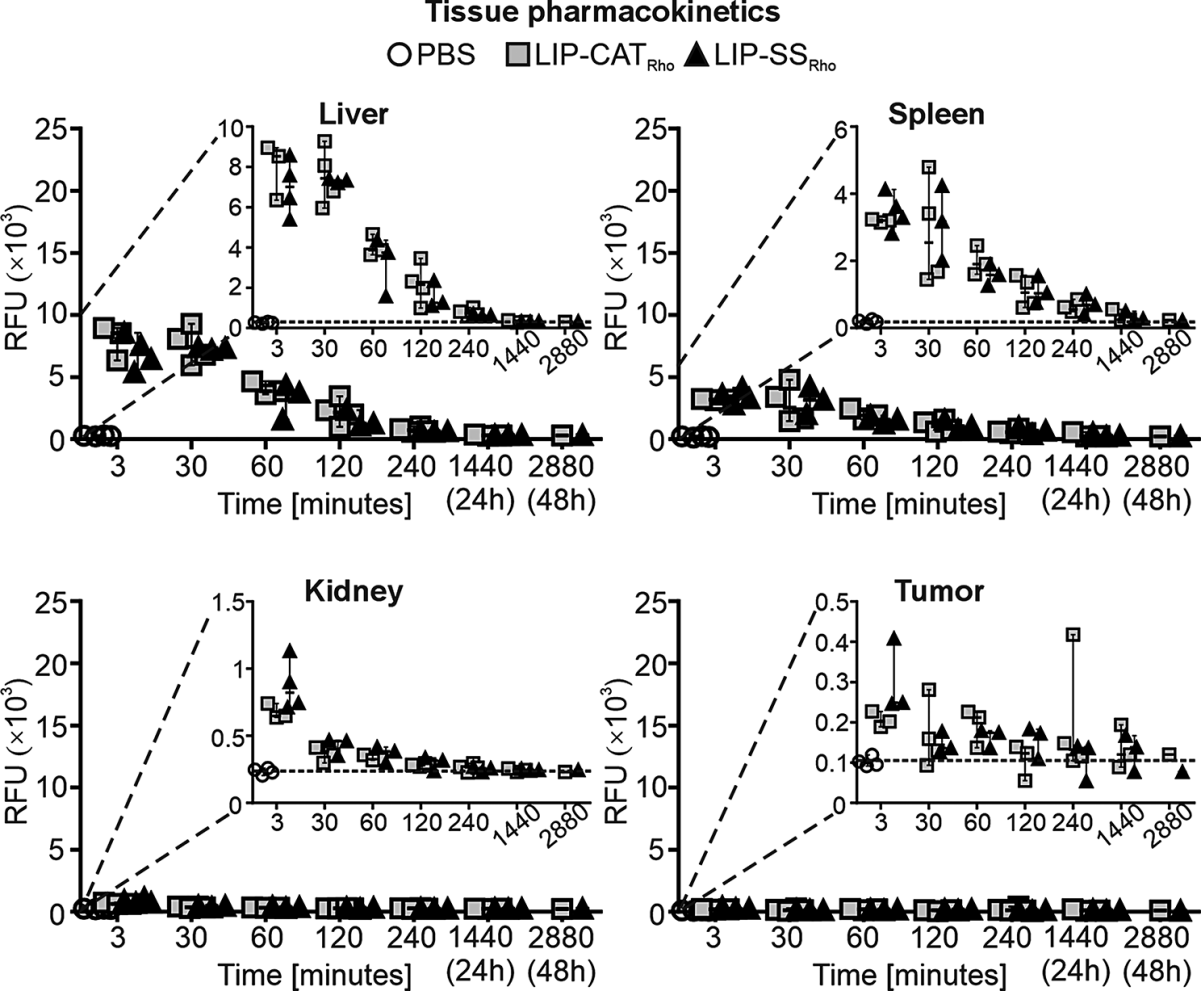
LIP-SS_Rho_ have identical tissue pharmacokinetics as LIP-CAT_Rho._ Fluorescently labeled LIP-CAT_**Rho**_, LIP-SS_**Rho**_, or PBS (negative control) were injected intravenously into the mice. Animals were euthanized at different time points after injection, and isolated organs and tumors were homogenized. Fluorescence measurements were performed for tissue homogenates using a microplate reader (excitation 560 nm; emission 590 nm). Points represent the data obtained in different experiments (*n* = 1–4)

The biodistribution studies were performed on mice bearing 4T1 orthotopic breast tumors. A comparison of the pharmacokinetics of intravenously injected, fluorescently labeled LIP-SS_Rho_ and LIP-CAT_Rho_ demonstrated that the silicone-stabilized liposomes have identical pharmacokinetic profiles as unmodified liposomes. As shown in Fig. 1A, the highest fluorescence intensity was measured for the sera isolated from the blood taken 3 min after administration of liposomes (~ 18,000 RFU) and markedly decreased for samples obtained 30 (~ 3000 RFU) and 60 (~ 2000 RFU) minutes later. After 240 min, the serum fluorescence level stabilized and remained at the same low level until 48 h after injection of LIP-SS_Rho_ or LIP-CAT_Rho_. High fluorescence intensity between 3 and 30 min was measured for the liver (~ 7000–8000 RFU) and the spleen (~ 3000 RFU, respectively), indicating the short accumulation of liposomes in these organs. Then, we observed a faster fluorescence decrease in the liver and spleen between 30 and 240 min after liposome injection (Fig. 2). At the same time, 3 min after injection, the lowest fluorescence intensity was measured for the kidney (~ 700–800 RFU) and the tumor (~ 200–300 RFU) (Fig. 2). Additionally, we determined liposome elimination half-lives based on pharmacokinetic profiles obtained for serum and selected organs (Fig. 1B, Table S1). The time required for the LIP-CAT_Rho_ and LIP-SS_Rho_ to decrease to half of the starting dose was short and similar for the serum and liver. Interestingly, liposomes LIP-SS_Rho_ were cleared faster than LIP-CAT_Rho_ from the kidney (75.1 min compared to 120.5 min, respectively). However, this observation is subject to certain limitations resulting from complex calculations made during data analysis (lower coefficient of determination calculated for this data compared with other data sets). This underscores the crucial need for further studies to validate and expand upon these findings (see data analysis provided at 10.57903/UJ/WTMVKS). The slowest elimination of both types of liposomes was determined for the spleen (86.1 min for LIP-CAT_Rho_ and 88.6 min for LIP-SS_Rho_).

**Fig. 3:**
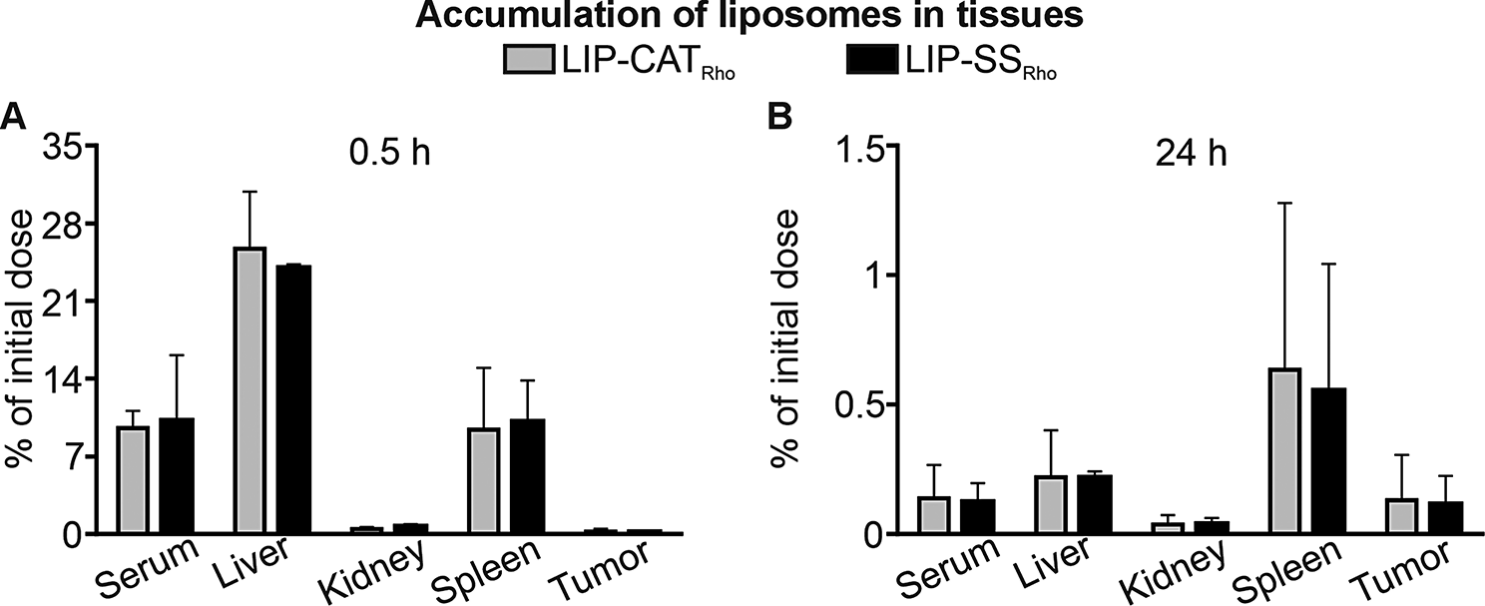
LIP-SS_Rho_ liposomes have identical biodistribution as LIP-CAT_Rho._ Fluorescently labeled LIP-CAT_Rho_, LIP-SS_Rho_, or PBS (negative control) were injected intravenously into the mice. Animals were placed in metabolic cages and euthanized **(A)** 0.5 h or **(B)** 24 h after injection. Isolated organs and tumors were homogenized. Fluorescence measurements were performed for tissue homogenates using a microplate reader (excitation 560 nm; emission 590 nm). Bars represent the mean ± SD (*n* = 3–4)

**Fig. 4:**
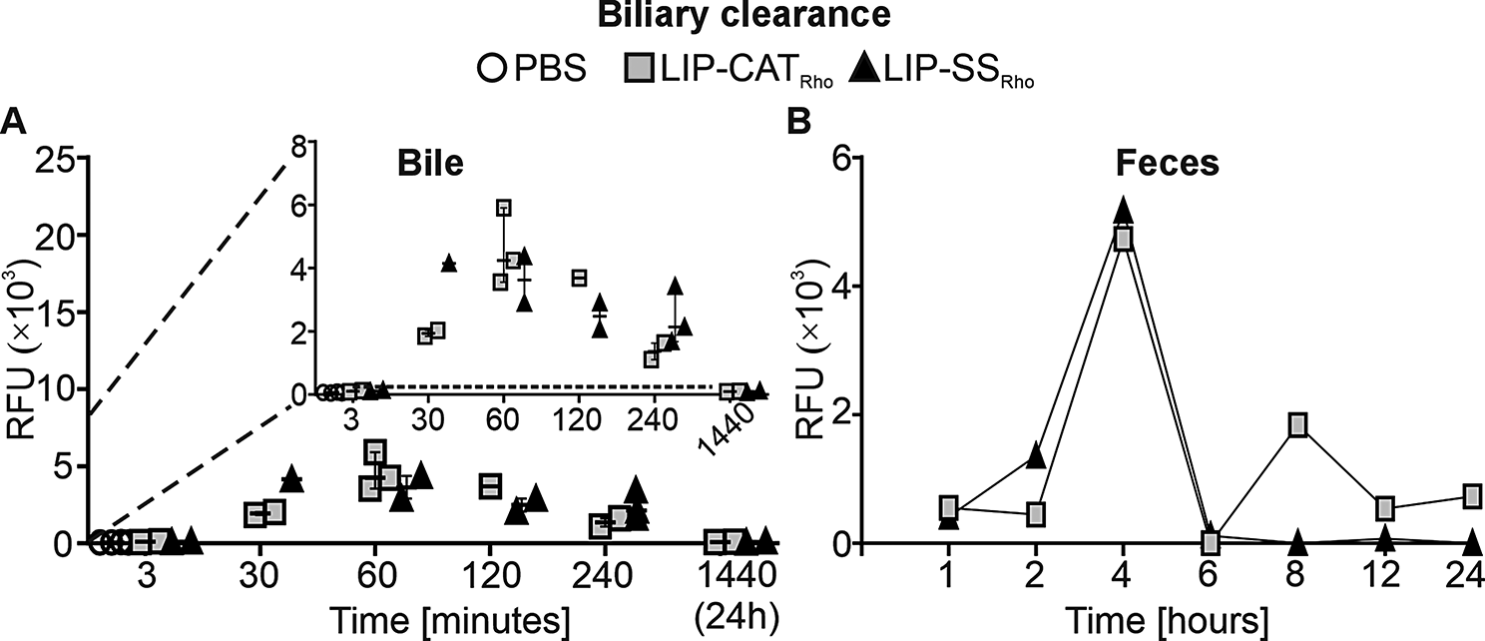
LIP-CAT_Rho_ and LIP-SS_Rho_ liposomes are eliminated from the body *via* hepatobiliary clearance. Fluorescently labeled LIP-CAT_Rho_, LIP-SS_Rho_, or PBS (negative control) were injected intravenously into the mice. Animals were placed in metabolic cages and euthanized at different time points after injection. (**A**) Bile was isolated (points represent the data obtained in different experiments; *n* = 1–4), and (**B**) feces were collected, weighted, and resuspended in PBS. Points represent the data obtained in different experiments (*n* = 2–3). Fluorescence measurements were performed for bile and feces suspensions using a microplate reader (excitation 560 nm; emission 590 nm)

The biodistribution of liposomes was analyzed using metabolic cages, allowing the collection of urine and feces and, thus, analysis of liposome elimination routes. We have observed that 30 min after injection, liposomes were still detectable in the serum (~ 10% of the initial dose) and accumulated predominantly in the liver (~ 25% of the initial dose) and in the spleen (~ 10% of the initial dose) (Fig. 3). Simultaneously, increased bile fluorescence intensity was measured with a maximum amount of ~ 4000 RFU 60 min after injection. This observation suggests that hepatocytes participated extensively in liposome uptake and elimination *via* hepatobiliary clearance (Fig. 4A). This hypothesis was supported by results obtained from feces collection; the highest fluorescence intensity was detected in the feces collected 4 h after administration (Fig. 4B).

**Fig. 5:**
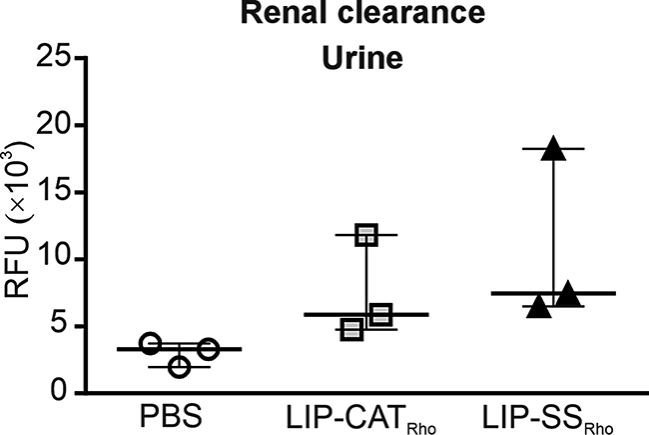
LIP-CAT_Rho_ and LIP-SS_Rho_ liposomes are also eliminated from the body *via* renal clearance Fluorescently labeled LIP-CAT_Rho_, LIP-SS_Rho_, or PBS (negative control) were injected intravenously into the mice. Animals were placed in metabolic cages. Urine was collected for 24 h, and fluorescence measurements were performed using a microplate reader (excitation 560 nm; emission 590 nm). Points represent the data obtained in different experiments (*n* = 3)

After 24 h, small amounts of LIP-SS_Rho_ and LIP-CAT_Rho_ were still detectable in selected tissues. The highest % of the initial dose was found in the spleen (~ 0.6% of the initial dose) and in the liver (~ 0.2% of the initial dose). The accumulation of liposomes in the tumor was consistent and low (~ 0.1% of the initial dose) at 30 min and 24 h after injection (Fig. 3). As presented in Fig. 5, fluorescence measurement performed for collected urine revealed that LIP-SS_Rho_ and LIP-CAT_Rho_ were also eliminated *via* renal clearance.

### The silicone network fabricated within the LIP-SS bilayer does not increase liposome toxicity compared to LIP-CAT

**Fig. 6:**
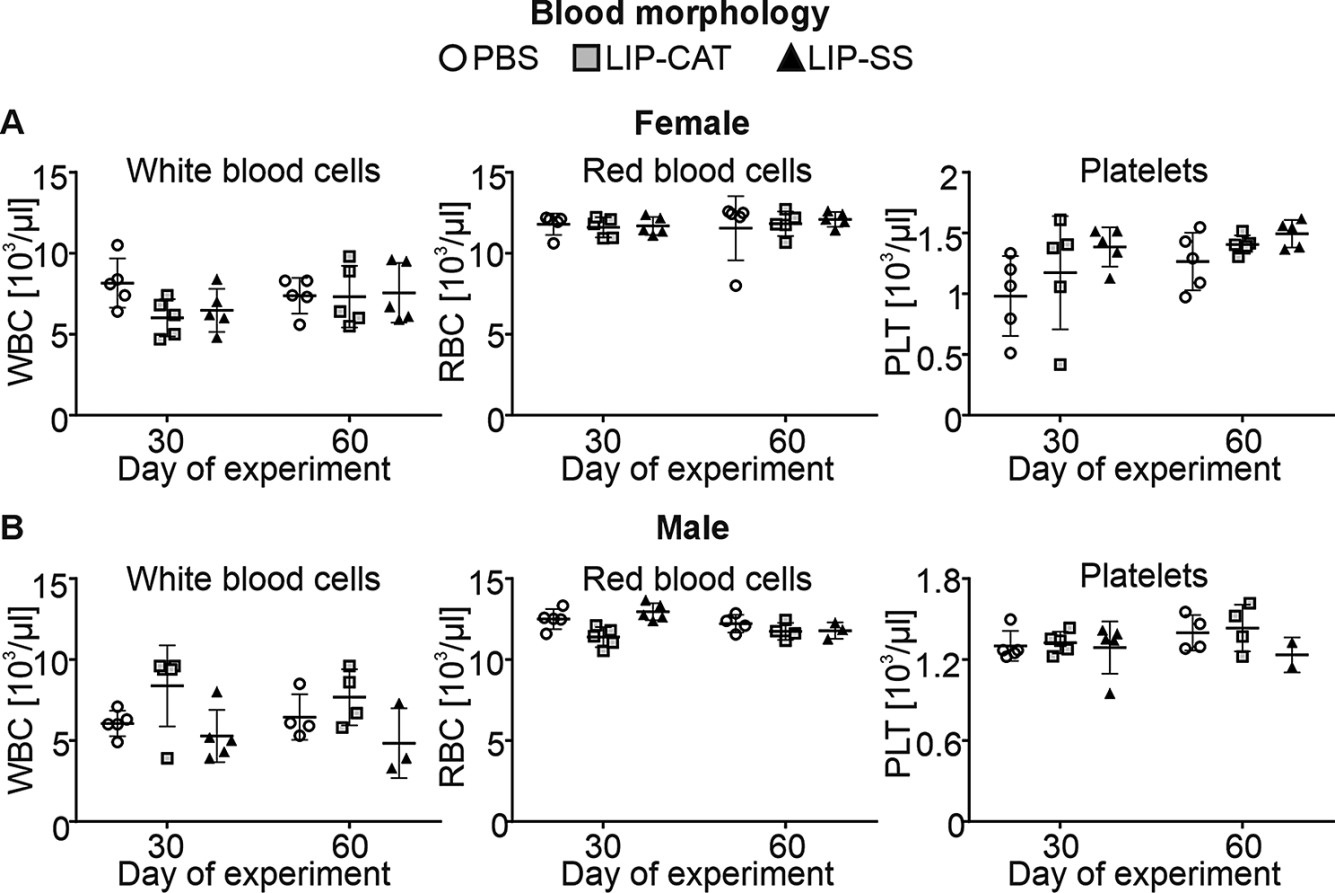
Blood morphology was analyzed for mice exposed to LIP-CAT, LIP-SS, or PBS (negative control). BALB/c mice (**A.** female and **B.** male) were exposed to 8 intravenously injected liposomes, LIP-CAT, LIP-SS, or PBS. The following day after the last dose (30th day of the experiment) or 30 days after the last dose (60th day of the experiment), animals were euthanized. Before euthanasia, blood for morphology analysis was taken from the facial vein. Analysis was performed using an ABC vet analyzer. Points represent the data obtained in different experiments (*n* = 2–5)

**Fig. 7:**
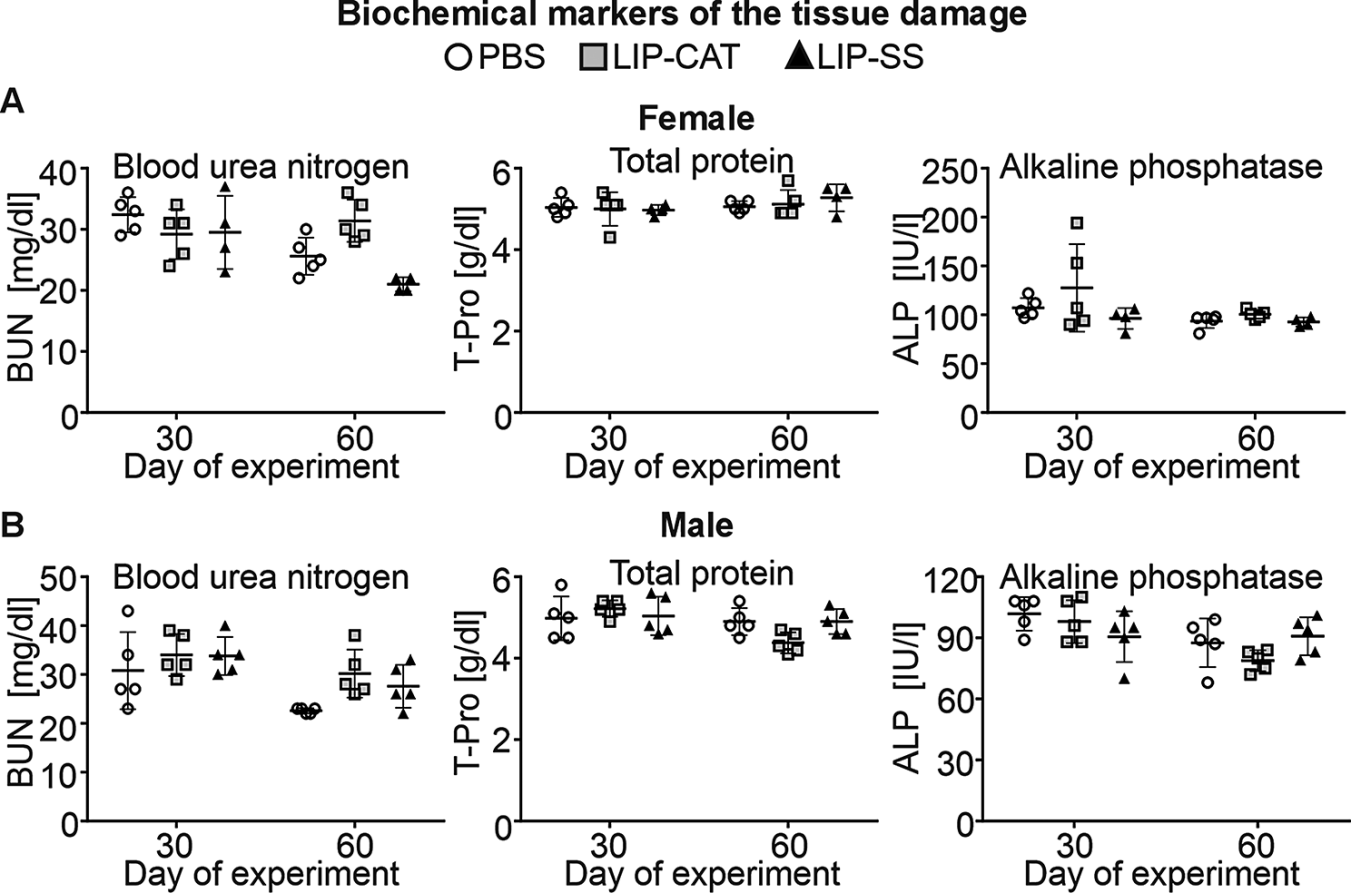
Tested liposomes are not hepato- or nephrotoxic for the animals – analysis of selected biochemical markers. BALB/c mice (**A.** female and **B.** male) were exposed to 8 intravenously injected liposomes, LIP-CAT, LIP-SS, or PBS. The following day after the last dose (30th day of the experiment) or 30 days after the last dose (60th day of the experiment), animals were euthanized. Blood for biochemical analysis was taken from the facial vein. Analysis was performed using isolated sera and the Spotchem EZ analyzer. Points represent the data obtained in different experiments (*n* = 4–5)

**Fig. 8:**
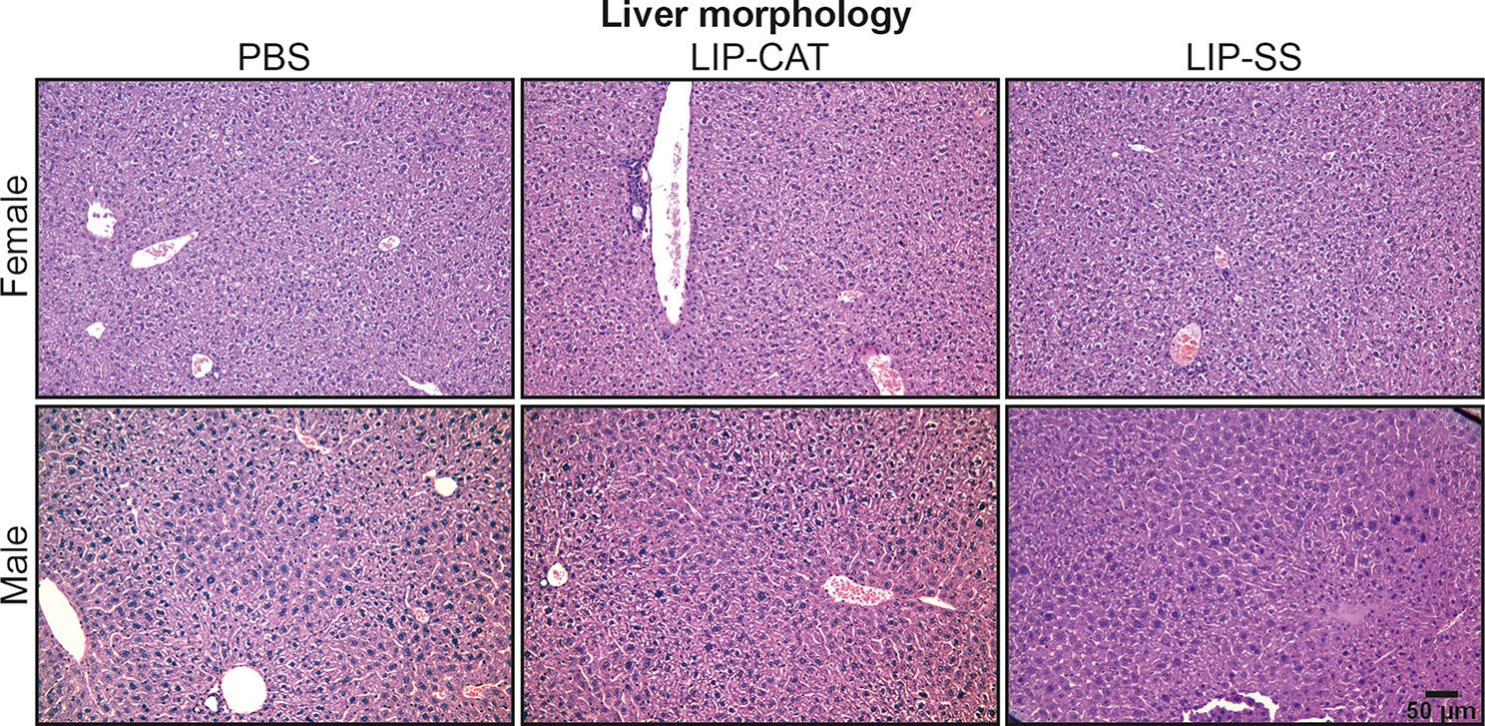
Tested liposomes do not exert toxicity against the liver – histopathological analysis. BALB/c mice (female and male) were exposed to 8 doses of intravenously injected liposomes LIP-CAT, LIP-SS, or PBS. Animals were euthanized on the 30th day after the last dose (60th day of the experiment). Livers were isolated, fixed, paraffine-embedded, stained, and subjected to histological analysis

**Fig. 9:**
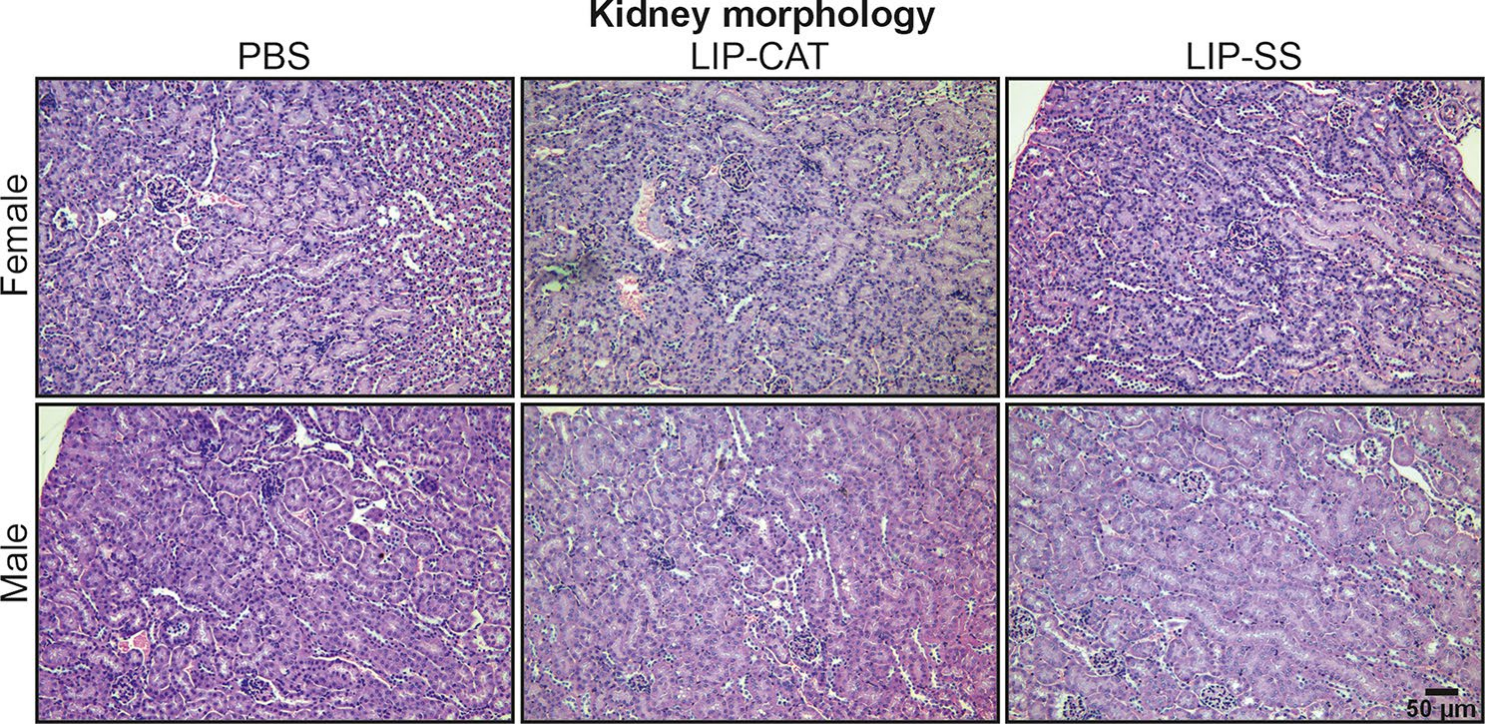
Tested liposomes do not exert toxicity against the kidney – histopathological analysis. BALB/c mice (female and male) were exposed to 8 doses of intravenously injected liposomes LIP-CAT, LIP-SS, or PBS. Animals were euthanized on the 30th day after the last dose (60th day of the experiment). Kidneys were isolated, fixed, paraffine-embedded, stained, and subjected to histological analysis

**Fig. 10:**
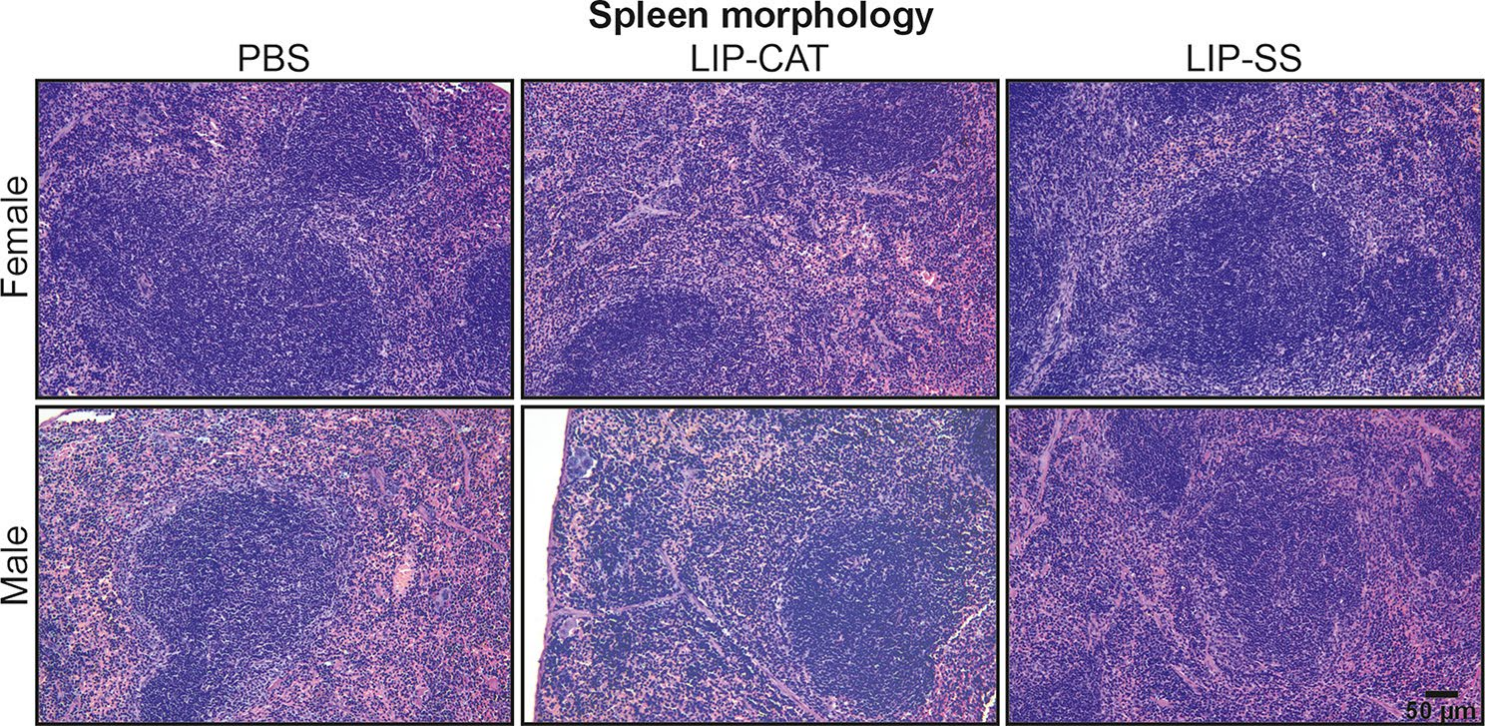
Tested liposomes do not exert toxicity against the spleen – histopathological analysis. BALB/c mice (female and male) were exposed to 8 doses of intravenously injected liposomes LIP-CAT, LIP-SS, or PBS. Animals were euthanized on the 30th day after the last dose (60th day of the experiment). Spleens were isolated, fixed, paraffine-embedded, stained, and subjected to histological analysis

**Fig. 11:**
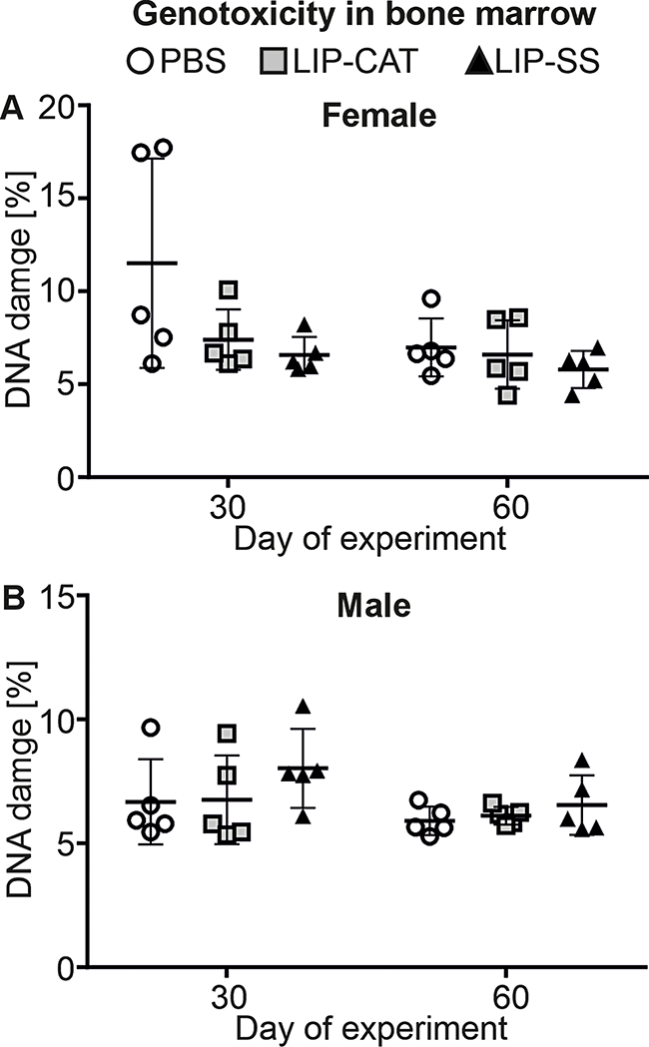
Tested liposomes are not genotoxic – analysis of DNA damage in the cells isolated from bone marrow. BALB/c mice (**A**. female and **B**. male) were exposed to 8 doses of intravenously injected liposomes LIP-CAT, LIP-SS, or PBS. The following day after the last dose (30th day of the experiment) or 30 days after the last dose (60th day of the experiment), animals were euthanized. Bone marrow cells were isolated from the femurs. Next, DNA damage was analyzed using a comet assay. Points represent the data obtained in different experiments (*n* = 5)

Before in vivo toxicity analysis, we tested endotoxin (LPS, lipopolysaccharide) contamination in liposome solutions. We evaluated the LPS level at 2.1, 6.8, or 9.2 EU/ml for PBS used for liposome synthesis, LIP-CAT, and LIP-SS, respectively (Figure S3). At the same time, we observed that these liposome solutions did not stimulate either mouse monocyte-macrophages (RAW 264.7) to produce NO – hallmark of inflammation or human TLR-4, TLR-2, or TLR-5 overexpressed in NF-ĸB-SEAP HEK293 reporter cells (Figure S4 and S5). The toxicity of LIP-SS and LIP-CAT were analyzed on healthy mice (female and male). The studies were designed to detect acute but primarily chronic toxicity of liposomes. Animals received eight doses of liposome intravenously and were euthanized the next day after the last injection (30th day) or 30 days after injecting the last dose of eight liposome doses (60th day). Blood morphology analysis performed for animals exposed to LIP-SS or LIP-CAT just before euthanasia revealed no changes compared to the control animals (Fig. 6 and S6). Similarly, we did not observe increased concentration or activity of hepatotoxicity (total protein, alkaline phosphatase, alkaline transaminase) or nephrotoxicity (blood urea nitrogen, creatinine) markers (Fig. 7 and S7). Moreover, histopathological evaluation of liver, kidney, and spleen supported results indicating biocompatibility of LIP-SS and LIP-CAT. No pathological changes were observed in the collected tissues either after hematoxylin and eosin staining or after SEM observation (Figs. 8 and 9, and 10, S8). Finally, a comet assay was performed for cells isolated from the bone marrow and confirmed that LIP-CAT and LIP-SS liposomes did not lead to DNA damage (Fig. 11).

### LIP-SS and LIP-CAT liposomes interact with the immune system, manifesting in upregulated production of cytokines, including proinflammatory ones

**Fig. 12:**
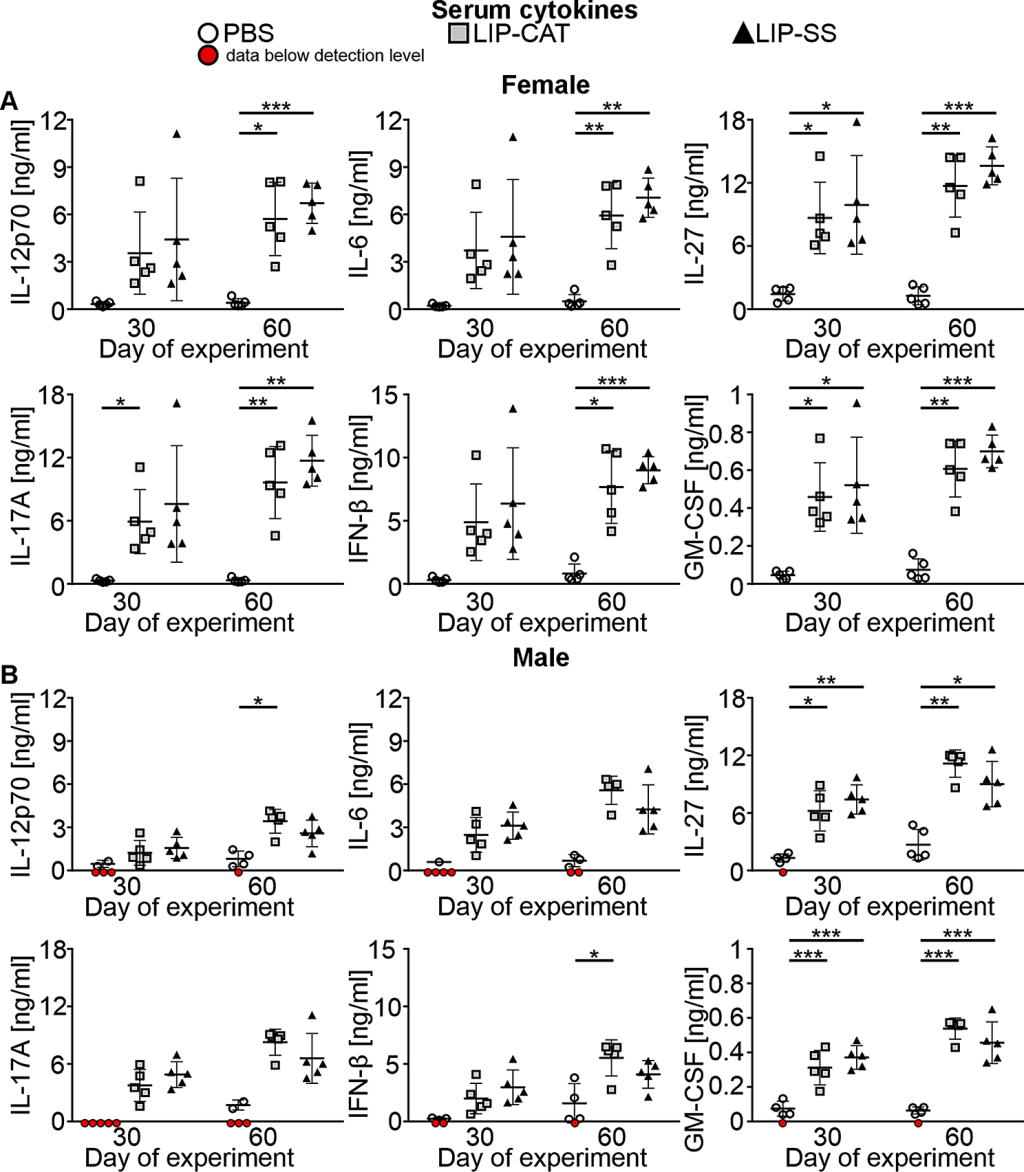
Intravenous administrations of LIP-CAT and LIP-SS lead to increased levels of proinflammatory cytokines in the sera. BALB/c mice (**A.** female and **B.** male) were exposed to 8 doses of intravenously injected liposomes LIP-CAT, LIP-SS, or PBS. The following day after the last dose (30th day of the experiment) or 30 days after the last dose (60th day of the experiment), animals were euthanized. Blood for sera isolation was taken by cardiac puncture. Analysis was performed using isolated sera, flow cytometry, and the Cytokine Biolegend Kit. The mean ± SD was calculated for points representing data obtained from different experiment replications within the detection range (*n* = 1–5). Red dots indicate that the experiment replicates were below the detection range.* *p* < 0.05; ** *p* < 0.01; *** *p* < 0.001

**Fig. 13:**
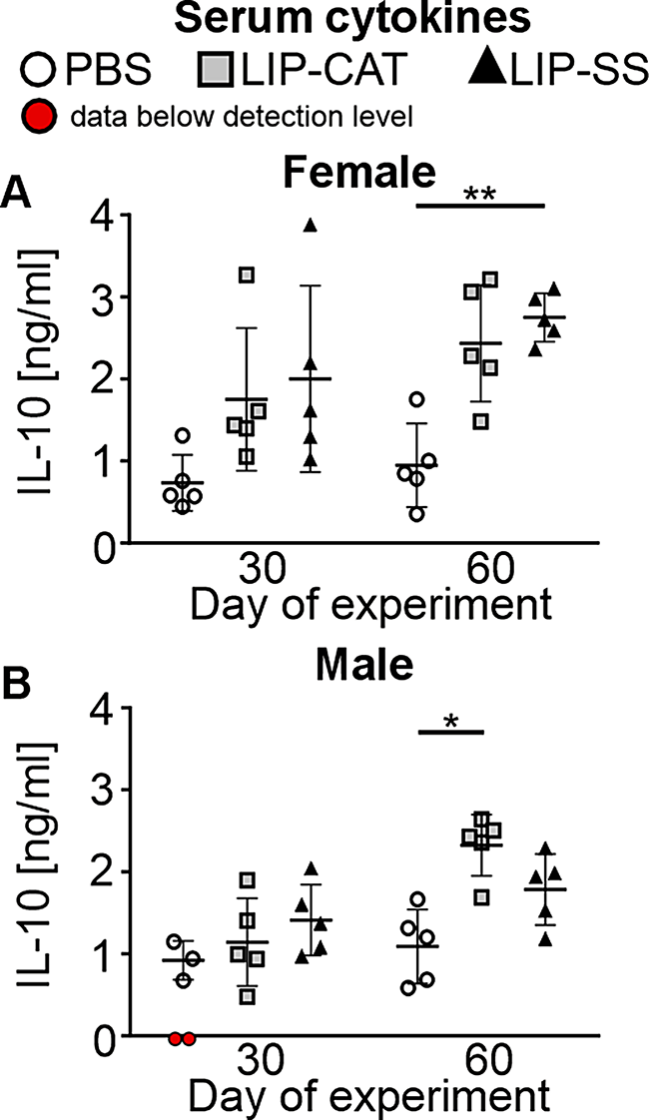
Intravenous administrations of LIP-CAT and LIP-SS lead to moderately increased levels of anti-inflammatory IL-10 in the sera. BALB/c mice (**A**. female and **B**. male) were exposed to 8 doses of intravenously injected liposomes LIP-CAT, LIP-SS, or PBS. The following day after the last dose (30th day of the experiment) or 30 days after the last dose (60th day of the experiment), animals were euthanized. Blood for sera isolation was taken by cardiac puncture. Analysis was performed using isolated sera, flow cytometry, and the Cytokine Biolegend Kit. The mean ± SD was calculated for points representing data obtained from different experiment replications within the detection range (*n* = 3–5). Red dots indicate that the experiment replicates below the detection range. * *p* < 0.05; ** *p* < 0.01

In the next step, we verified the interaction of liposomes with the immune system by cytokine analysis in the mice sera. We observed that animals exposed to LIP-SS but also to LIP-CAT liposomes had upregulated levels of numerous cytokines, including interleukins (IL-23, IL-1α, IL-12p70, IL-1β, IL-10, IL-6, IL-27, IL-17 A), interferons (IFN-β, IFN-γ), TNF-α, chemokine MCP-1 and GM-CSF on the 30th day of the experiment. Moreover, for almost all cytokines, no concentration decrease was observed in sera collected on the 60th day of the experiment, thus, as many as 30 days after the last liposome injection (Figs. 12 and 13, and S9). The most significant increase was revealed for IL-12p70, IL-6, IL-27, IL17A, IFN-β, and GM-CSF (Fig. 12) and the smallest for IL-10 (Fig. 13). In addition, we did not notice elevated levels of IFN-γ, TNF-α, and IL-6 in the serum of BALB/c mice with 4T1 tumors after 1, 2, 4, or 24 h of liposome injection (Figure S10). Therefore, these results indicate prolonged activation of the immune system.

## Discussion

### Liposome stabilization *via* the silicone layer does not affect their biodistribution profile

The analysis of biodistribution and pharmacokinetics of nanoparticles, including liposomes, has some limitations. In most cases, additional nanomaterial labeling is required to visualize nanoparticles in vivo. Examples of nanoparticle labeling agents include isotopes, fluorophores, and ferromagnetics. Moreover, the results of nanoparticle biodistribution could be determined by the use of specific markers, the labeling method, and the nanoparticle compartment chosen for labeling [20-22). At the same time, the labeling agent can be both hydrophilic and hydrophobic, allowing the staining of an aqua or lipid space of liposomes. All this makes comparing the biodistribution results of different groups difficult and complex.

Our previous work demonstrated better elastomechanical properties (higher Derjaguin–Muller–Toporov modulus) of silicone-stabilized liposomes than unmodified liposomes. We also observed a trend for a faster uptake of rigid LIP-SS by peripheral blood mononuclear cells after the first hour of adding the liposomes to the cell culture medium [18]. Another group also demonstrated higher cellular internalization of stiff liposomes by non-mucus-producing Caco-2 cells 23].

We have previously shown that the release of calcein from the aqueous space of liposomes is much slower for LIP-SS than for LIP-CAT, confirming the better in vitro stability of LIP-SS [17]. Therefore, in this pharmacokinetic and biodistribution study, we decided to use a lipid space marker to avoid results affected by premature leakage of the hydrophilic fluorophore from tested liposomes. We demonstrated a similar DMT modulus for fluorescently labeled liposomes compared to unlabeled ones (Figure S2). Results presented in this work revealed no differences in pharmacokinetic and biodistribution profiles between liposomes differing in elastomechanical properties. Other researchers also observed no significant differences in the elasticity-dependent accumulation of intravenously injected nanoparticles in mice healthy tissues [12,15]. Additionally, we noticed a short serum half-life (~ 60 min) of liposomes, which may be insufficient to demonstrate enhanced permeability and retention effect 24]. The tested liposomes’ low accumulation (~ 0.1% of the initial dose) in tumor tissue, regardless of their elastic properties, confirms this hypothesis. Contrary to our findings, another group’s results showed that the accumulation of liposomes with different Young’s modulus in the 4T1 tumor depends on nanoparticle elasticity [25]. However, there may be several reasons for this discrepancy between the results of our study and Guo et al. We employed L-α-phosphatidylethanolamine-N-(lissamine B sulfonyl rhodamine) – Liss Rho PE to label liposomes, and fluorescence was measured after tissue homogenization. In contrast, Guo et al. used the lipophilic carbocyanine DiOC_18_ and measured fluorescence in whole mice or tissues using the IVIS Lumina II system. Reports indicate that Liss Rho PE, more than other liposome labeling probes, undergo transport *via* scavenger receptor B-I. Since this receptor is expressed on hepatic cells, it can drive the elimination of Liss Rho PE to the bile [26,27]. Therefore, we suggest that the Liss Rho PE we chose may increase the liver accumulation of liposomes, affecting distribution to other tissues, including tumors.

Our study demonstrates that silicone stabilization alone does not improve the pharmacokinetic profiles of liposomes or ensure their superior tumor accumulation compared to unmodified liposomes. We reached this conclusion by evaluating the half-lives of liposomes in plasma, which indirectly reflects their in vivo stability. Interestingly, we found no significant difference in the plasma half-life of LIP-SS and LIP-CAT, suggesting that liposomes’ in vivo stability is similar despite their in vitro stability differences [17]. This implies that the behavior of liposomes, including their interaction with serum proteins, blood cells, hepatocytes, Kupfer cells, etc., remains consistent regardless of their elastomechanical properties. Therefore, our study suggests that improving liposomes’ elastomechanical properties without simultaneous surface functionalization with hydrophilic stealth polymers is insufficient to achieve long-circulating liposomes. We imply that the mechanism of rapid clearance of all tested liposomes is related to their removal by the hepatobiliary clearance supported by the reticuloendothelial system [21]. Consequently, we postulate liposomes LIP-SS require further surface modification to achieve long-circulation properties.

### Stimulation of immune response does not result from potential LPS contamination of liposome samples

Our studies revealed that both liposome types led to the upregulation of various cytokines, including standard cytokines produced by immune cells in response to LPS, namely IL-6, TNF, IL-1β, and MCP-1. However, LPS contamination measured for liposome samples (~ 7–9 EU/ml) using the Limulus Amebocyte Lysate assay did not exceed the concentration suggested as acceptable by the FDA (Figure S3). According to the FDA recommendation, the approximate threshold pyrogen dose for humans and rabbits for intravenous exposure is 5 EU/kg of body mass. In addition, this threshold is divided by the maximum human dose per kilogram that would be administered in one hour, so endotoxin limits are different for each drug 29]. Since we injected 0.2 ml of liposomes solution per mouse, the LPS amount in one dose was ~ 70–90 EU/kg (~ 10 ng/kg). Therefore, to safely use the tested liposomes in humans, we must either prepare a solution with a lower level of endotoxin or use a lower dose. Since mice are much less sensitive to endotoxin than humans, we do not assume that the cytokine overproduction observed in mice sera is solely related to LPS contamination [30]. This hypothesis is confirmed by our results on RAW 264.7 cells, which neither under normal conditions nor in the presence of the immune response enhancer - IFN-γ were stimulated by liposome solutions. We also observed that liposomes did not modulate the response of RAW 246.7 cells to LPS (Figure S4). Additionally, we used a model susceptible to pathogen-associated molecular patterns (PAMPs) - NF-ĸB-SEAP HEK293 reporter cells overexpressing human TLR4, TLR2/1, TLR2/6, or TLR5. These receptors recognize LPS, CU-T12-9 (synthetic small-molecule agonist), FSL-1 (synthetic lipoprotein), and flagellin, respectively. We have shown that the liposome solution is not recognized by any tested receptors (Figure S5). Therefore, we assume that the impurity concentrations in liposome solutions are insufficient for activating NF-ĸB by TLRs. Finally, we showed no overproduction of IFN-γ, TNF-α, and IL-6 in the serum of mice shortly after administration of liposomes (Figure S10). Serum levels of these pro-inflammatory cytokines peak 2–7 h after exposure to LPS and decrease after that [31]. The fact that we did not observe IFN-γ, TNF-α, and IL-6 after 1, 2, and 4 h of liposome exposure suggests that the effect of long-term cytokine overproduction is due to repeated administration of liposomes rather than endotoxin contamination.

Our in vivo studies showed that tested liposomes, modified with a silicone layer and control liposomes, have immunostimulant properties manifesting in the upregulation of cytokines, including proinflammatory ones. Prolonged activation of the immune system and elevated levels of cytokines increase the risk of inflammation-mediated toxicity. TNF-α is a major proinflammatory cytokine upregulated in chronic inflammatory and autoimmune diseases. However, other cytokines, IFN-γ, IL-23, IL-6, IL-17α, and GM-CSF, can also play a role in these pathologies [32]. IL-23 is the cytokine essential for inducing differentiation of CD4 + T cells into T helper cells that secrete IL-17 (Th17), a key proinflammatory cytokine involved in the pathogenesis of T-cell mediated autoimmune diseases. Recently published data revealed that lung exposure to inhaled particulate matter (PM2.5) could lead to IL-17 A signaling pathway upregulation and increased TGF-β production, the major inductor of chronic lung injuries [33]. Moreover, interleukin-23 may induce the proliferation of cancer cells, promoting tumor growth and development. At the same time, increased IL-23 and IL-17 were associated with a poor prognosis of non-small cell lung cancer [34]. Interestingly, our results demonstrated an upregulated concentration of anti-inflammatory IL-10 cytokine; however, IL-10 concentration was the slightest increase among other tested serum cytokines (Fig. 13). The question arises as to whether appropriate anti-inflammatory mechanisms might overcome the effect induced by the overproduction of liposome-derived proinflammatory cytokines.

Unfortunately, we do not know the mechanism of the observed phenomenon indicating long-term toxicity manifesting in increased cytokines production. Particularly worrying is that the concentration of the tested cytokines is high after the last administration, which has been a long time. Similar results, indicating upregulated cytokine production, were previously obtained by our team for intravenously injected biodegradable polyelectrolyte nanocapsules functionalized with PEG. Interestingly, as for LIP-CAT and LIP-SS, after administration of the nanocapsules functionalized with PEG, we observed no evidence of toxicity of the nanocarriers other than elevated levels of serum cytokines [35]. There is still a limited amount of data on cytokines production in response to nanomaterials, particularly biodegradable, and its clinical relevance. Therefore, further studies should be performed to elucidate the mechanism of this phenomenon.

## Conclusions and study limitations

In this paper, we characterized the in vivo behavior of silicone-stabilized liposomes for the first time. Our results showed no differences between tested liposomes in the context of their pharmacokinetics, biodistribution, and tumor accumulation. We, therefore, assume that the better elastomechanical properties of silicone-stabilized liposomes are insufficient to modulate liposome in vivo behavior significantly, and further surface modifications are necessary to extend their circulation time. Moreover, we presented the immunomodulatory properties of the tested liposomes after repeated intravenous administration. The elevated level of cytokines in serum was long-lasting, indicating undesired activation of immune cells exposed to tested liposomes. Therefore, it is essential to investigate the mechanisms of this phenomenon. Our results are of crucial importance in the field of nanoparticle design and their applications as drug delivery systems. At the same time, we acknowledge the limitations of our research. We investigated two types of liposomes that differ significantly in terms of elastomechanical properties. However, it would be necessary to prepare a comprehensive library of liposomes to unambiguously analyze the influence of elastomechanical properties on the behavior of these nanomaterials in vivo. This library could include liposomes that differ only in elastomechanical parameters. We also suggest that before library construction, it is necessary to functionalize the liposome surface to ensure their long circulation in the plasma.

### Electronic supplementary material

Below is the link to the electronic supplementary material.

Supplementary Material 1

## Data Availability

The datasets generated and/or analyzed during the current study are available in the RODBUK repository: https://doi.org/10.57903/UJ/WTMVKS.
