# Supplementary material for: The elasticity of silicone-stabilized liposomes has no impact on their in vivo behavior
Source: J Nanobiotechnology. 2024 Aug 5;22:467. doi: 10.1186/s12951-024-02698-9 (PMC11299312; doi:10.1186/s12951-024-02698-9)
Supplement: Supplementary file 1 — Supplementary Material 1 [file 12951_2024_2698_MOESM1_ESM.docx]

Supplementary materials


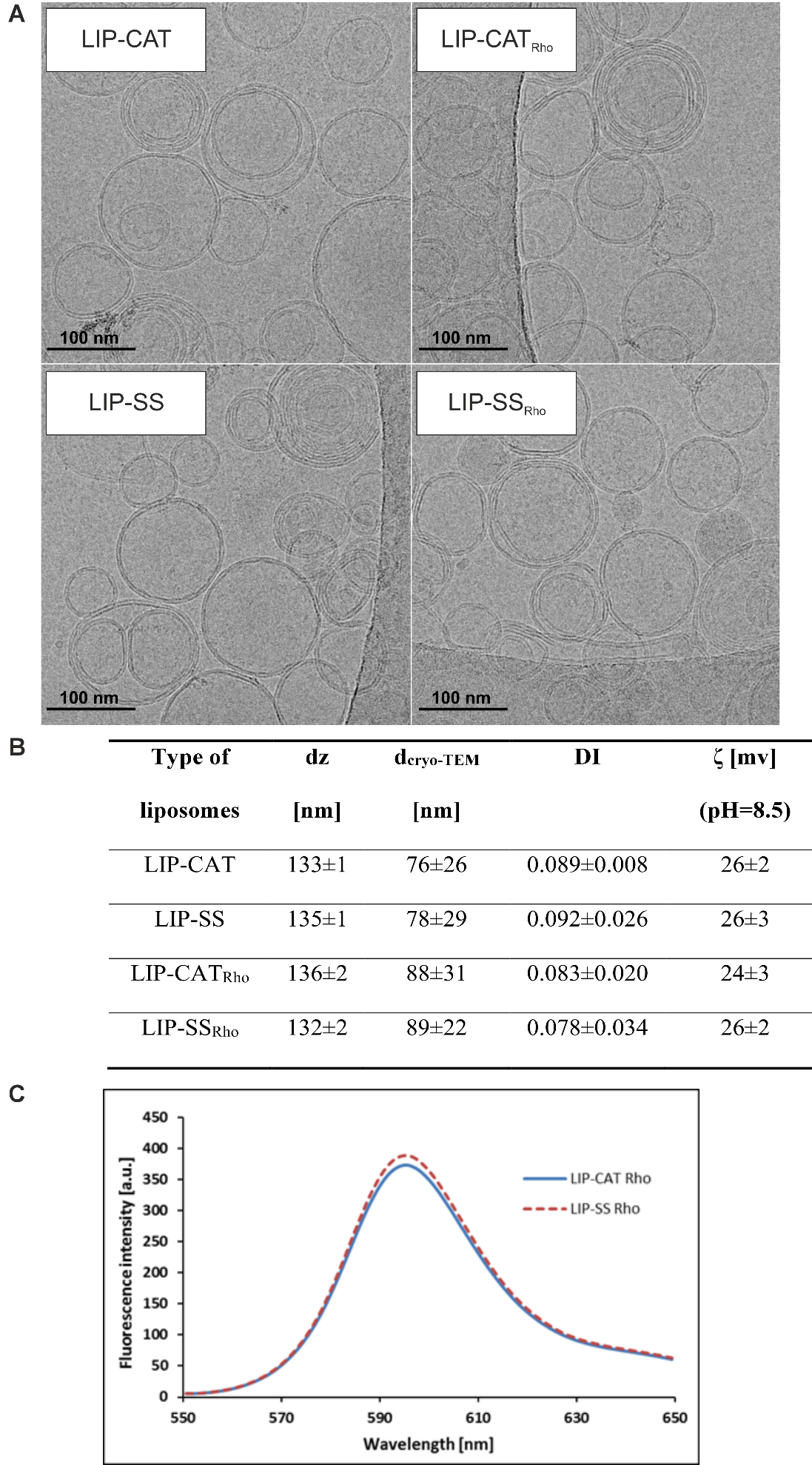


**Figure S1.** **A**. Cryo-TEM images of liposomes (LIP-CAT, LIP-SS) and liposomes fluorescently labeled (LIP-CAT_Rho_ and LIP-SS_Rho_). **B.** The values of mean hydrodynamic diameter (dz), values of the mean diameter calculated based on cryo-TEM microphotographs (d_cryo-TEM_±SD), dispersity index (DI) and zeta potential (ζ) for LIP-CAT, LIP-SS and liposomes fluorescently labelled with PE-Rhod, LIP-CAT_Rho_ and LIP-SS_Rho_, respectively. **C.** Fluorescence spectra of LIP-CAT_Rho_ and LIP-SS_Rho_ (λ_exc_ = 560 nm).


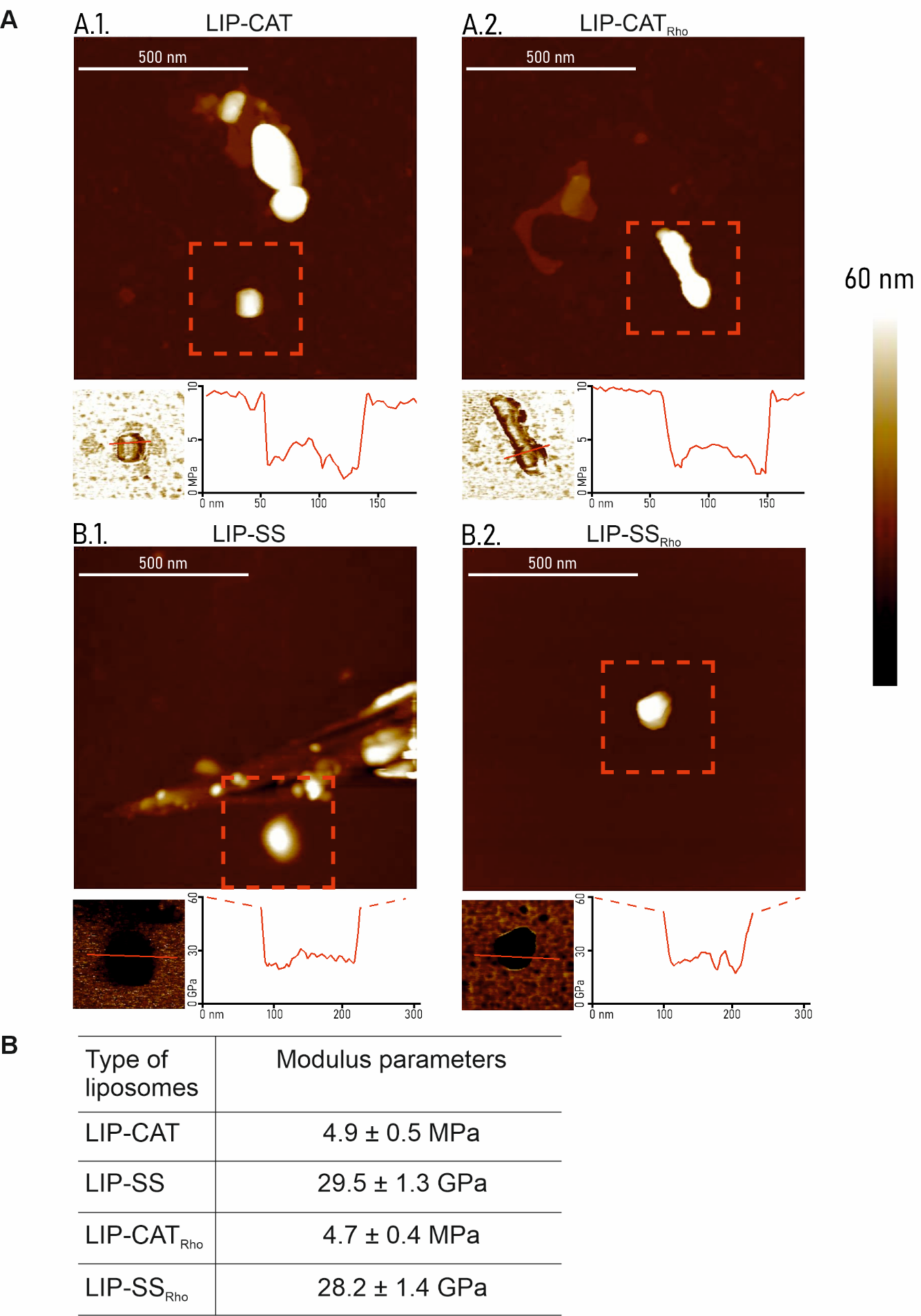


**Figure S2.** **A.** Atomic Force Microscopy topography and Derjaguin–Muller–Toporov (DMT) modulus images of liposomes (LIP-CAT, LIP-SS) and liposomes fluorescently labeled (LIP-CAT_Rho_ and LIP-SS_Rho_) **B**. Calculated modulus parameters (av. from 12 lines for each liposome).

**
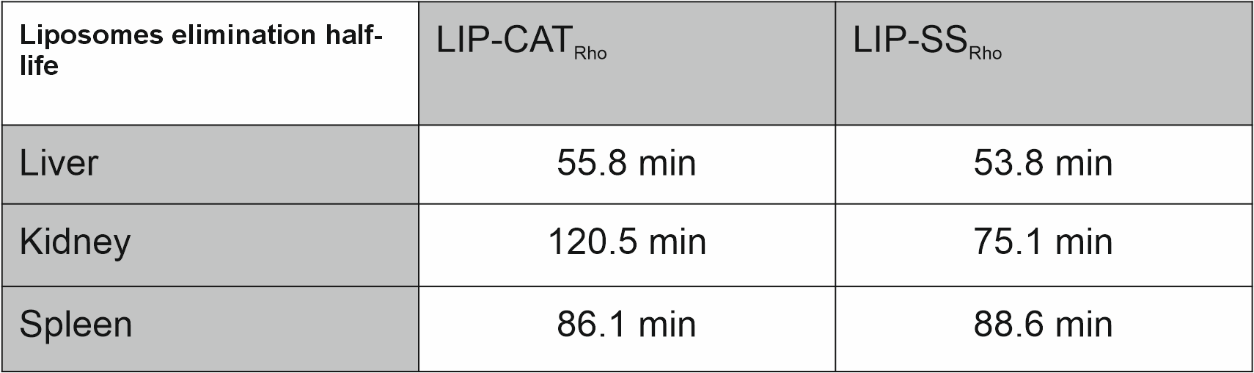
**

**Table S1. Liposome elimination half-life.** The elimination half-life was calculated using pharmacokinetic profiles in the selected organs determined after intravenous administration of fluorescently labeled liposomes LIP-CAT_Rho_ and LIP-SS_Rho_.


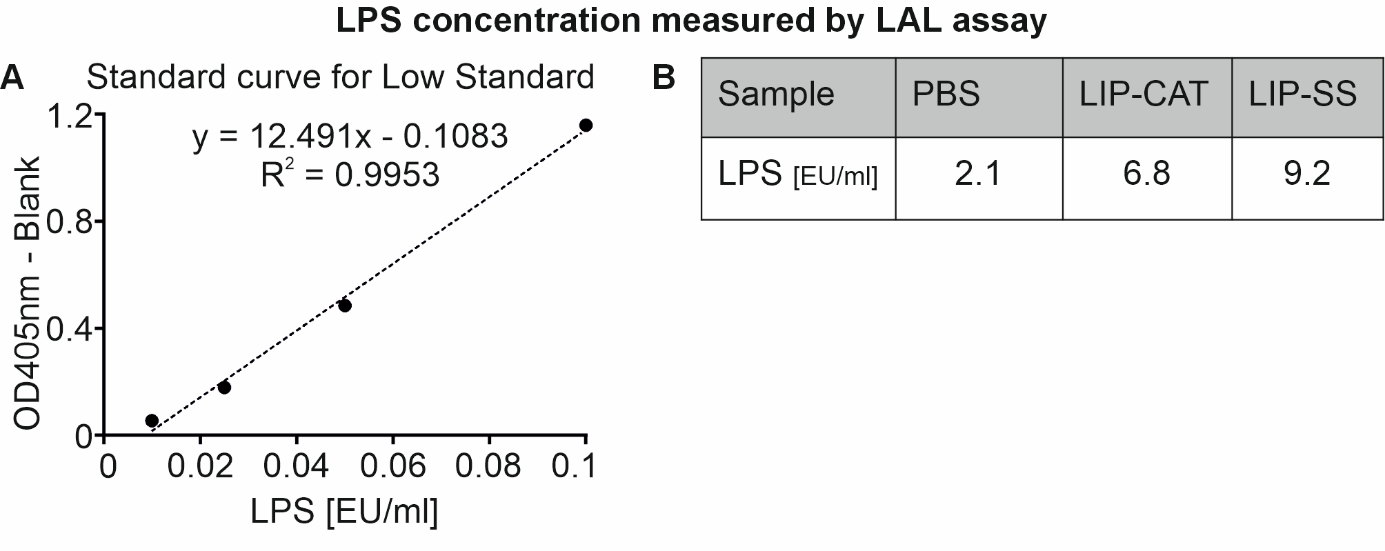


**Figure S3. Detection of LPS in samples of liposomes used for *in vivo* studies.** The level of LPS in samples was analyzed using the Limulus lysate assay (LAL).


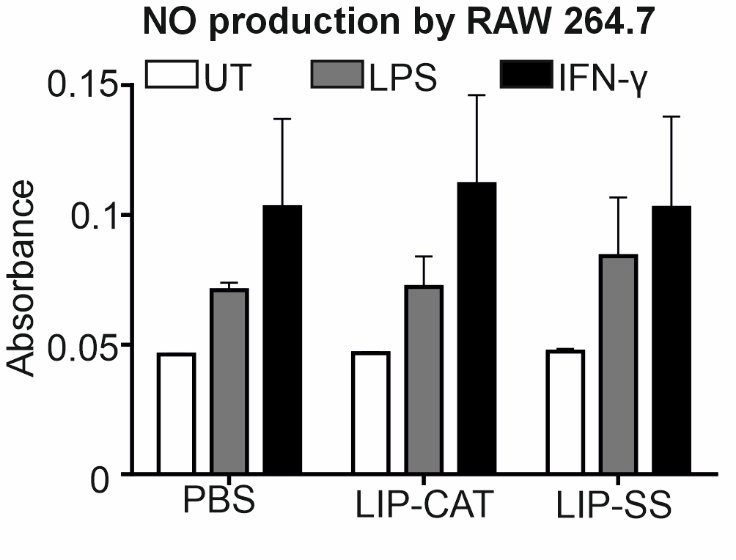


**Figure S4. Analysis of NO production by inducible nitrate oxide synthase.** Cell culture media was harvested from RAW 264.7 after 24 hours of incubation with liposomes in the absence (UT - untreated) or in the presence of LPS (100 ng/ml) or IFN-γ (10 ng/ml). Nitrite levels were measured by Griess reaction and presented as absorbance.


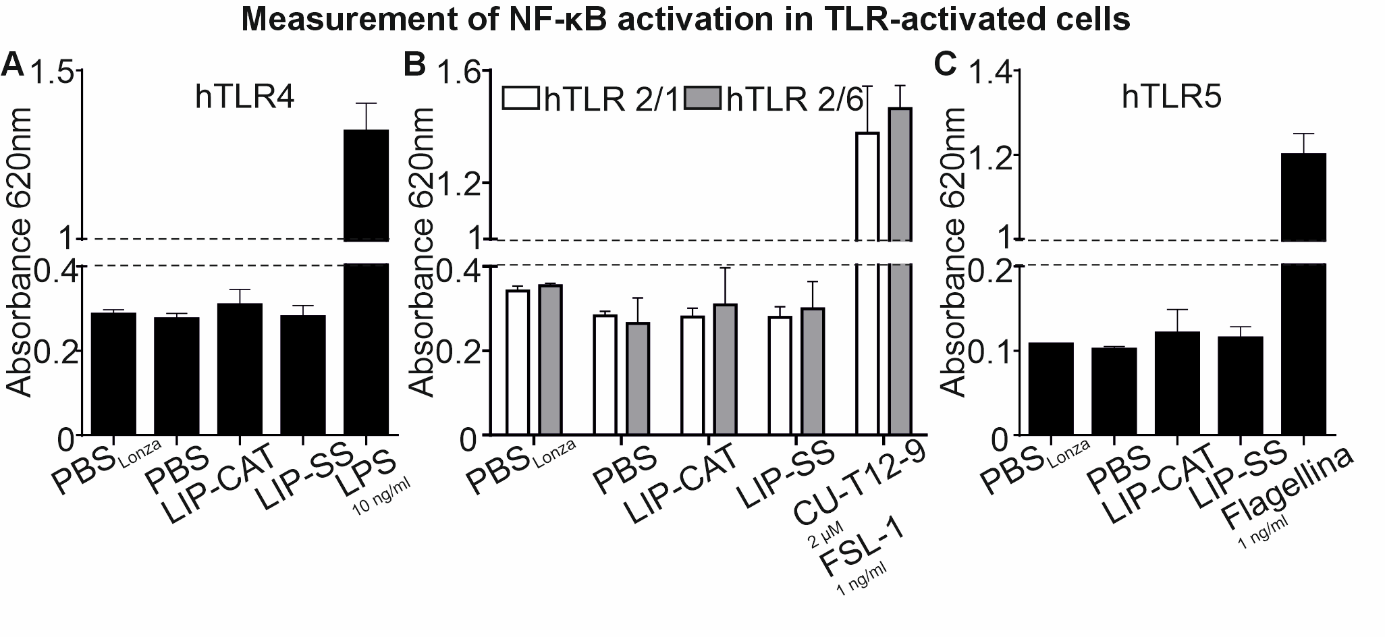


**Figure S5. Interaction of LIP-CAT and LIP-SS with TLR receptors.** Analysis was performed using HEK–blue reporter cells expressing hTLR4, hTLR2/TLR1, hTLR2/TLR6, and hTLR5. Activation of TLRs expressed on reporter cells results in NF-ĸB activation and Secretory Embryonic Alkaline Phosphatase expression (SEAP). The activity of secreted SEAP to the culture media indicates TLR activation. PBS_Lonza_ stands for PBS used for synthesizing liposomes before pH adjustment (LPS contamination measured for this solution was below the detection limit of the LAL test).


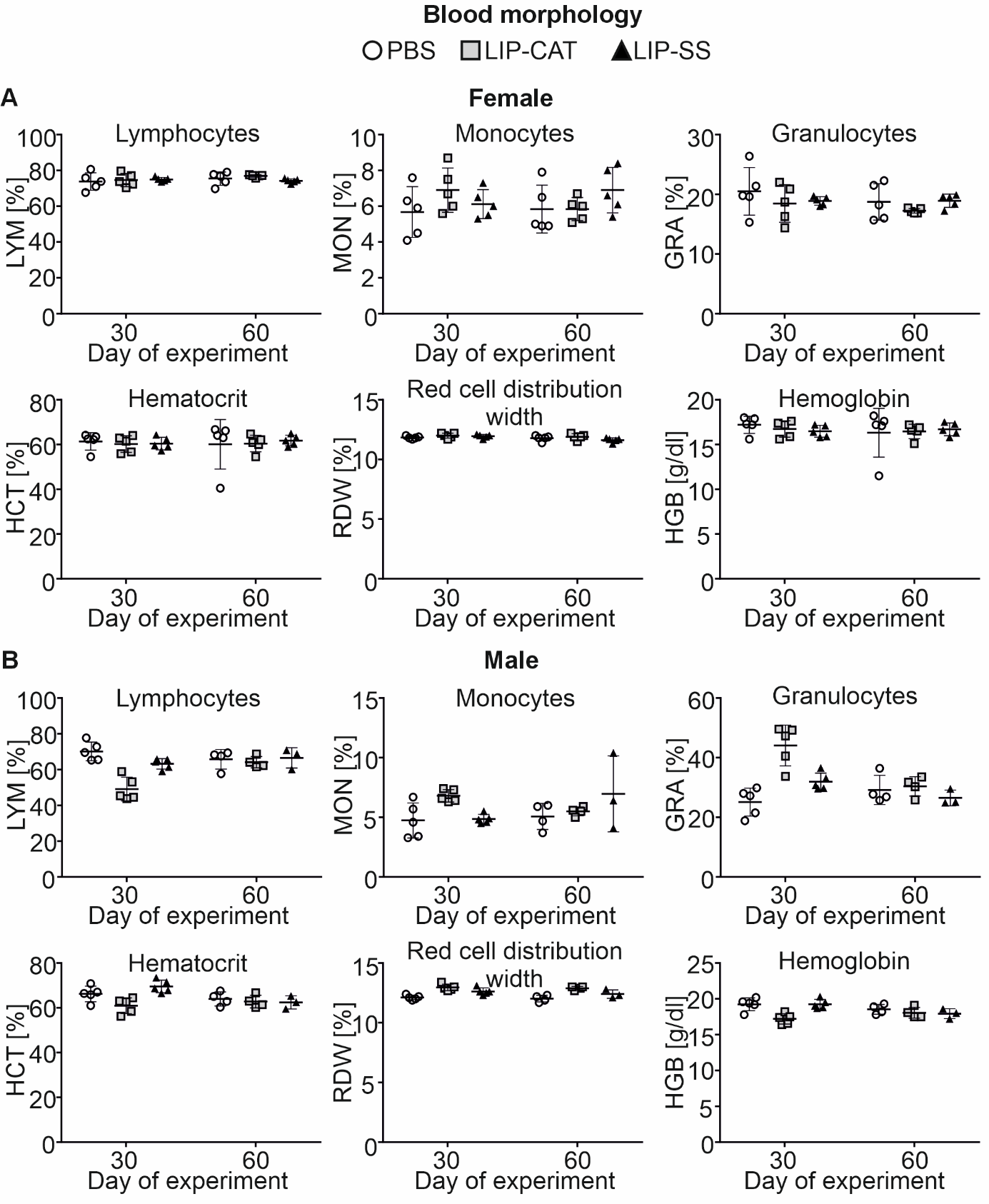


**Figure S6. Blood morphology was analyzed for mice exposed to LIP-CAT, LIP-SS, or PBS (negative control).** BALB/c mice (**A.** female and **B.** male) were exposed to 8 doses of intravenously injected liposomes or PBS. The following day after the last dose (30^th^ day of the experiment) or 30 days after the last dose (60^th^ day of the experiment), animals were euthanized. Before euthanasia, blood for morphology analysis was taken from the facial vein. Analysis was performed using an ABC vet analyzer.


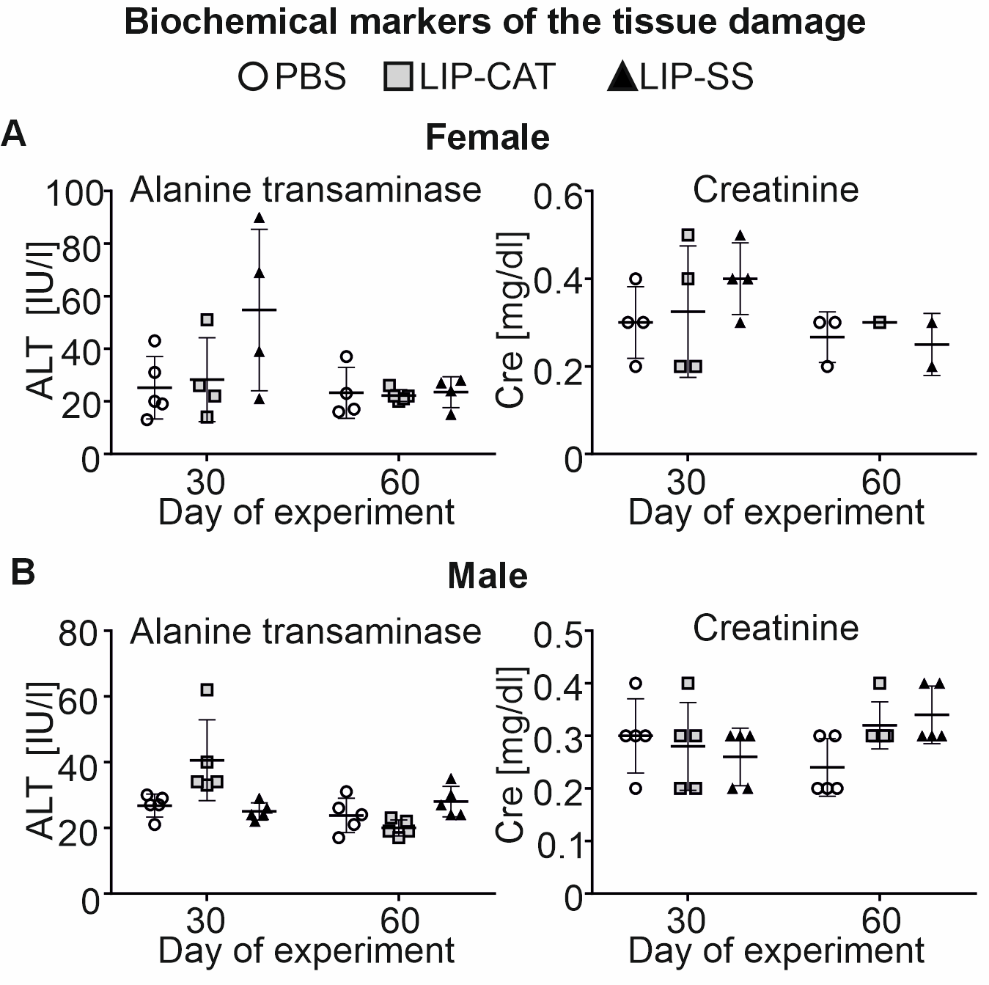


**Figure S7. Tested liposomes are not hepato- or nephrotoxic for the animals – analysis of selected biochemical markers.** BALB/c mice (**A.** female and **B.** male) were exposed to 8 doses of intravenously injected liposomes LIP-CAT, LIP-SS, or PBS. The following day after the last dose (30^th^ day of the experiment) or 30 days after the last dose (60^th^ day of the experiment), animals were euthanized. Blood for biochemical analysis was taken from the facial vein. Analysis was performed using isolated sera and the Spotchem EZ analyzer.


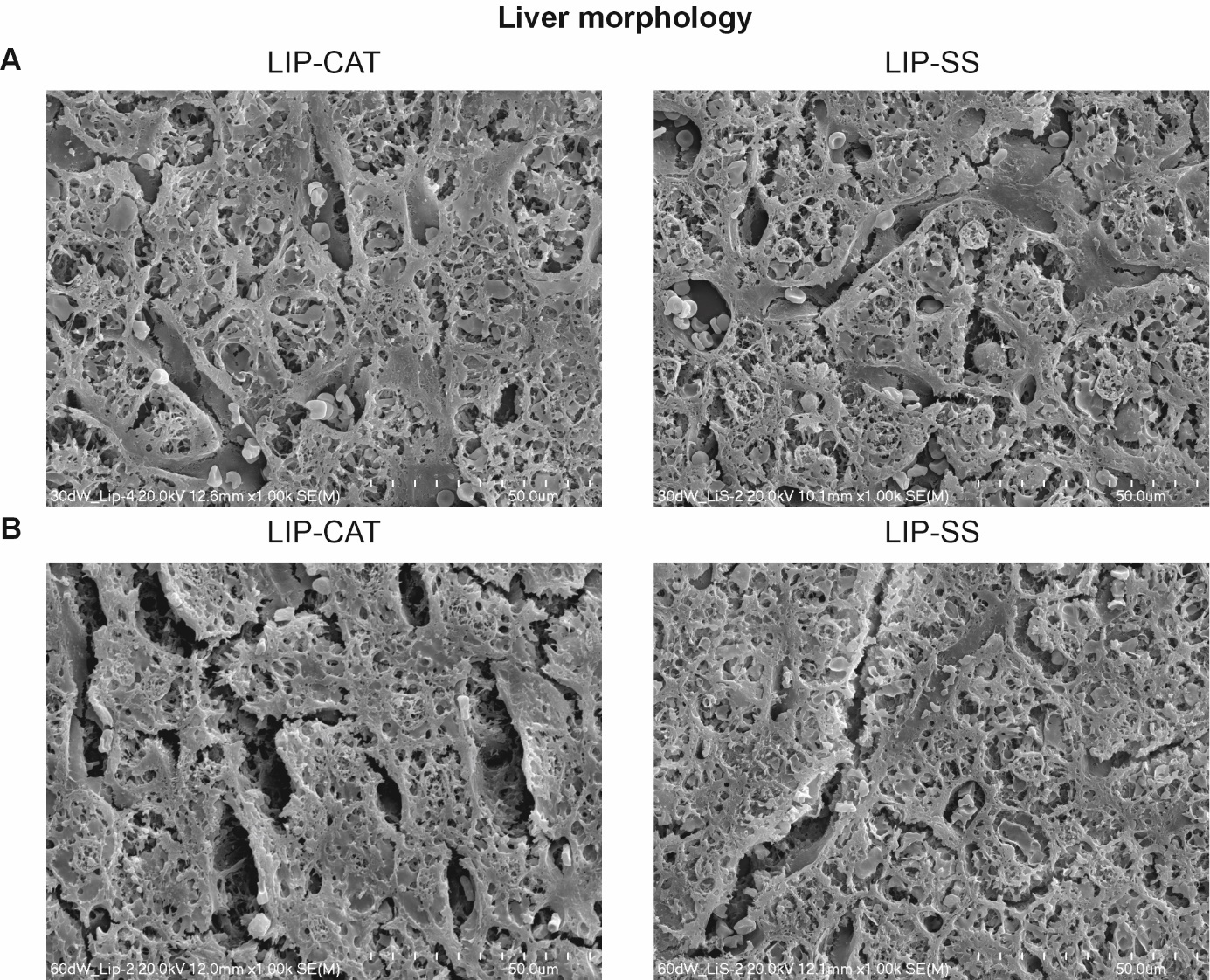


**Figure S8. Scanning electron microscope *(*SEM) microphotographs of liver tissue collected after multiple liposome exposures.** BALB/c mice (female) were exposed to 8 doses of intravenously injected liposomes LIP-CAT, LIP-SS, or PBS. The following day after the last dose (**A.** 30^th^ day of the experiment) or 30 days after the last dose (**B.** 60^th^ day of the experiment), animals were euthanized. Livers were isolated, fixed, **paraffin-embedded,** and subjected to SEM analysis.


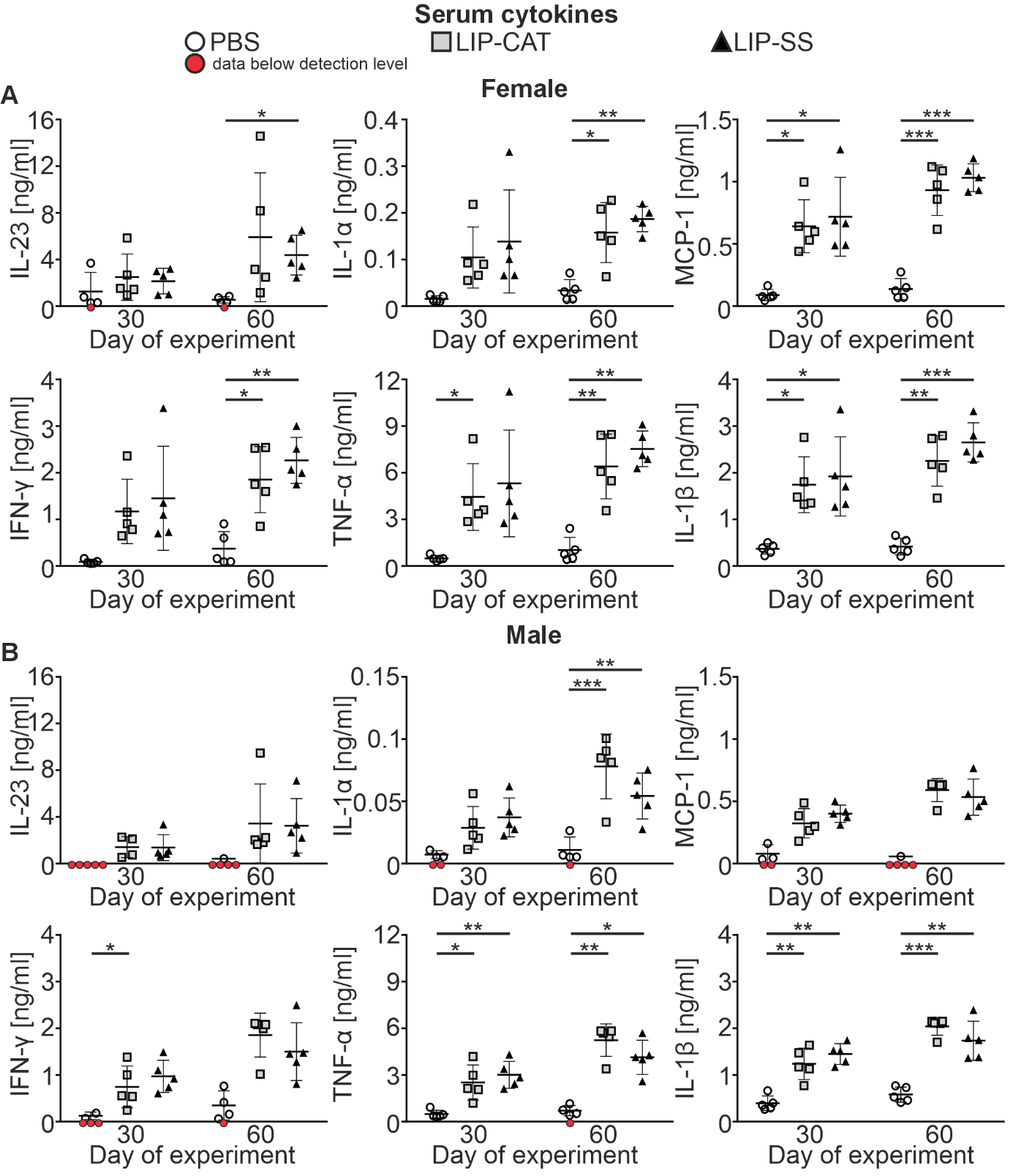


**Figure S9. Intravenous administrations of LIP-CAT and LIP-SS lead to increased levels of proinflammatory cytokines in the sera.** BALB/c mice (**A.** female and **B.** male) were exposed to 8 doses of intravenously injected liposomes LIP-CAT, LIP-SS, or PBS. The following day after the last dose (30^th^ day of the experiment) or 30 days after the last dose (60^th^ day of the experiment), animals were euthanized. Blood for sera isolation was taken by cardiac puncture. Analysis was performed using isolated sera, flow cytometry, and the Cytokine Biolegend Kit. The mean ± SD was calculated for points representing data obtained from different experiment replications within the detection range. Red dots indicate that the experiment replicates were below the detection range. * p < 0.05; ** p < 0.01; *** p < 0.001


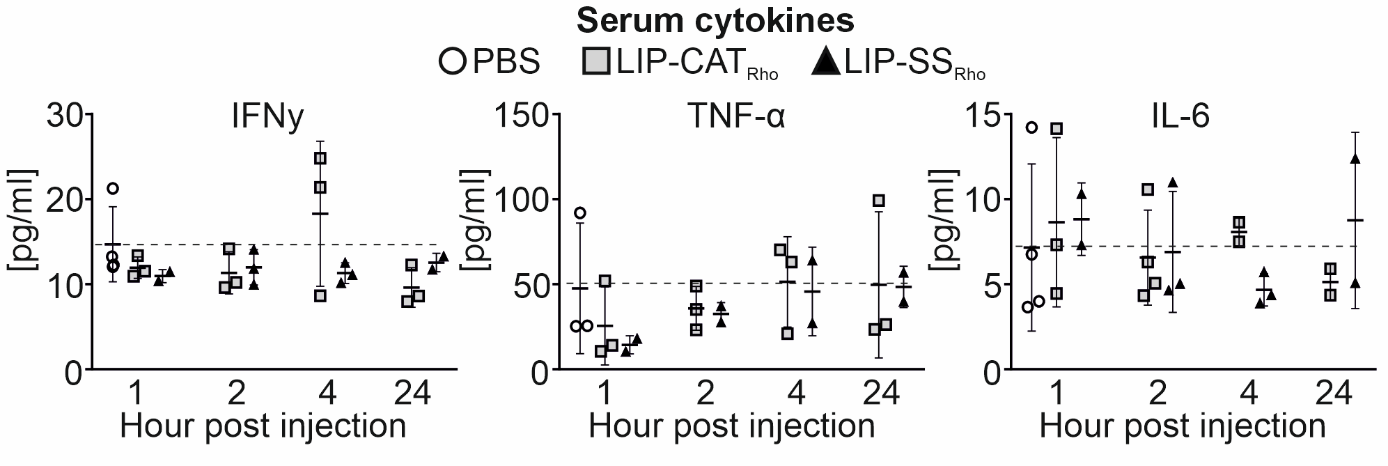


**Figure S10. Levels of cytokines in the mice's sera after single liposome exposure.** BALB/c mice with orthotopic breast cancer were injected intravenously with fluorescently labeled LIP-CAT_Rho_, LIP-SS_Rho_, or PBS. Blood for sera isolation was collected by cardiac puncture 1, 2, 4, or 24 h after injection. Analysis was performed using isolated sera, flow cytometry, and the Cytokine Biolegend Kit. The dashed line corresponds to the cytokine concentration measured for sera obtained from control mice injected with PBS.
